# Heterogeneity of Genetic Admixture Determines SLE Susceptibility in Mexican

**DOI:** 10.3389/fgene.2021.701373

**Published:** 2021-08-03

**Authors:** Susana Hernández-Doño, Juan Jakez-Ocampo, José Eduardo Márquez-García, Daniela Ruiz, Víctor Acuña-Alonzo, Guadalupe Lima, Luis Llorente, Víctor Hugo Tovar-Méndez, Rafael García-Silva, Julio Granados, Joaquín Zúñiga, Gilberto Vargas-Alarcón

**Affiliations:** ^1^Immunogenetics Division, Department of Transplant, Instituto Nacional de Ciencias Médicas y Nutrición Salvador Zubirán, Mexico City, Mexico; ^2^Department of Immunology and Rheumatology, Instituto Nacional de Ciencias Médicas y Nutrición Salvador Zubirán, Mexico City, Mexico; ^3^Molecular Biology Core Facility, Instituto Nacional de Enfermedades Respiratorias Ismael Cosío Villegas, Mexico City, Mexico; ^4^Department of Dermatology, Hospital General Dr. Manuel Gea González, Mexico City, Mexico; ^5^Laboratory of Physiology, Biochemistry, and Genetics, Escuela Nacional de Antropología e Historia, Mexico City, Mexico; ^6^Department of Endocrinology, Instituto Nacional de Ciencias Médicas y Nutrición Salvador Zubirán, Mexico City, Mexico; ^7^Department of Internal Medicine, Instituto Nacional de Ciencias Médicas y Nutrición Salvador Zubirán, Mexico City, Mexico; ^8^Laboratory of Immunobiology and Genetics, Instituto Nacional de Enfermedades Respiratorias Ismael Cosío Villegas, Mexico City, Mexico; ^9^Tecnologico de Monterrey, Escuela de Medicina y Ciencias de la Salud, Mexico City, Mexico; ^10^Research Direction, Instituto Nacional de Cardiología Ignacio Chavez, Mexico City, Mexico

**Keywords:** HLA, Mexican, population genetics, heterogeneity, HLA-DRB1^∗^03:01, admixture, conserved extended haplotype

## Abstract

Systemic Lupus Erythematosus (SLE) is an autoimmune inflammatory disorder for which Major Histocompatibility Complex (MHC) genes are well identified as risk factors. SLE patients present different clinical phenotypes, which are partly explained by admixture patterns variation among Mexicans. Population genetic has insight into the high genetic variability of Mexicans, mainly described through HLA gene studies with anthropological and biomedical importance. A prospective, case-control study was performed. In this study, we recruited 146 SLE patients, and 234 healthy individuals were included as a control group; both groups were admixed Mexicans from Mexico City. The HLA typing methods were based on Next Generation Sequencing and Sequence-Based Typing (SBT). The data analysis was performed with population genetic programs and statistical packages. The admixture estimations based on HLA-B and -DRB1 revealed that SLE patients have a higher Southwestern European ancestry proportion (48 ± 8%) than healthy individuals (30 ± 7%). In contrast, Mexican Native American components are diminished in SLE patients (44 ± 1%) and augmented in Healthy individuals (63 ± 4%). HLA alleles and haplotypes’ frequency analysis found variants previously described in SLE patients from Mexico City. Moreover, a conserved extended haplotype that confers risk to develop SLE was found, the HLA-A^∗^29:02∼C^∗^16:01∼B^∗^44:03∼DRB1^∗^07:01∼DQB1^∗^02:02, *p*C = 0.02, OR = 1.41. Consistent with the admixture estimations, the origin of all risk alleles and haplotypes found in this study are European, while the protection alleles are Mexican Native American. The analysis of genetic distances supported that the SLE patient group is closer to the Southwestern European parental populace and farthest from Mexican Native Americans than healthy individuals. Heterogeneity of genetic admixture determines SLE susceptibility and protection in Mexicans. HLA sequencing is helpful to determine susceptibility alleles and haplotypes restricted to some populations.

## Introduction

Systemic Lupus Erythematosus (SLE) is a chronic autoimmune disease characterized by the loss of tolerance to self-antigens and interferon responses dysregulation. SLE manifestations are diverse; the condition can affect almost any organ in the body (Ghodke-Puranik and Niewold, 2015). Patients with Hispanic, African, and Asian ancestry develop SLE earlier than European populations. These patients also have more acute disease onset, more severe clinical manifestations, higher disease activity, chronic organ damage, and higher mortality (Carter et al., 2016). Hispanic SLE prevalence is ∼138/100000 per inhabitants per year, which is higher than in Asian and European populations (Atisha-Fregoso et al., 2011). Many differences in the disease presentation across the ethnic barrier have been explained throughout genetic predisposition. One of the most studied systems is the Major Histocompatibility Complex (MHC) class I and class II genes, known as Human Leukocyte Antigen (HLA) Class I and II. The HLA variability among populations is helpful to explain many characteristics of SLE (Tsokos et al., 2016), and it continues to give more information about the genetic predisposition and pathophysiological mechanism of the disease.

As mentioned before, HLA haplotypes confer susceptibility or protection in an ethnic-dependent manner (Vargas-Alarcón et al., 2001; Vasconcelos et al., 2009; Furukawa et al., 2014; Alarcón-Riquelme et al., 2016; Molineros et al., 2019). In Mexico, susceptibility varies as the ethnic admixture does, the admixture in Mexico is very heterogeneous across the Country, contributing to the disease variability (Moreno-Estrada et al., 2014; Barquera et al., 2020b). Therefore, the admixture diversity has contributed to the enrichment of susceptibility markers and an ample SLE phenotypes specter in Mexicans (Salgado-Galicia et al., 2020). For instance, the most common susceptibility allele, HLA-DRB1^∗^03:01, previously identified in Mexico City, was not found in Tapachula Chiapas SLE patients. Otherwise, HLA-DR2 Chiapas patients showed a higher risk of developing SLE once infected with Zika or Chikungunya viruses common in that region but absent in Mexico City (Sepúlveda Delgado et al., 2018). Besides, population genetic studies revealed significantly different admixture estimates for Mexico City and Tapachula, Chiapas (Barquera et al., 2020e).

Equally important, conserved extended haplotypes (CEHs) help to defined susceptibility, and the alleles help MHC genetic diversity measurements in autoimmune conditions. CEHs are DNA blocks defined as combinations of HLA-B, -DR, complement, and other immune-related genes. CEHs are known as DNA stretches with fixed alleles, including those at loci not tested (Wescott et al., 1987). Thus, CEHs have a high genetic load, so determining HLA CEHs gives information about the aggregated risk confer by non-classical and non-HLA genes linked to HLA. The frequency and allele combination of CEHs varies between major ethnic groups. Hence, the determination of CEH and the ethnic admixture print allows knowing immunogenetic variants and blocks of anthropological origin and evolution but biomedical importance in Mexican mestizo patients.

Thus, this study aimed to describe SLE patients’ ethnic admixture proportions compared with healthy Mexican Mestizo individuals. We searched the distribution of HLA class I and class II blocks and CEH and their most likely ancestral origins using high-resolution HLA typing in a Mexican SLE group of admixed-ancestry.

## Subjects, Materials, and Methods

### Subjects

We recruited 146 consecutive SLE patients between 2015 and 2018 from the Rheumatology Outpatient Clinic at the Instituto Nacional de Ciencias Médicas y Nutrición Salvador Zubirán (INCMNSZ) in Mexico City. As a control group, 234 unrelated healthy Mexican admixed individuals were included. Eligible SLE patients included those born in Mexico, whose parents and grandparents were also born in Mexico. Specifically, those who were born in Mexico City and its borders. The same criterion was applied for controls.

The general health status was evaluated or investigated for both SLE and controls. The SLE patients’ diagnosis and classification were based on clinical manifestations, laboratory tests, and the American College of Rheumatology (ACR) criteria (Hochberg, 1997). The general health status in SLE patients was assessed with the SLE Activity Index (SLEDAI) (Bombardier et al., 1992; Guzmán et al., 1992). The patients were classified with punctuations between 0 and 4 it means inactive disease state or mild activity. The SLE classification punctuation was a criterion to establish the activity of the disease at the time of the interview.

Additionally, Systemic Lupus International Collaborating Clinics/American College of Rheumatology (SLICC/ACR) Damage Index (SDI) (Gladman et al., 2000) was registered for patients. This index was considered part of the health status evaluations, and it was thought as applicable to associate chronic evolution to genetic predisposition. The patients’ quality of life was also evaluated with the questionnaire Lupus Quality of Life (Lupus Qol), ranging in punctuations from 0 to 100 (Devilliers et al., 2012). Additionally, it was verified that all patients were consecutive in their medical controls and complete compliance with pharmacological treatment. All the above, to rule out that a severe phenotype, evolution at the study moment, or chronic damage was due to lack of accessibility to medical service, lack of patient adherence, additional stresses concerning the quality of life, and more likely associated with an immunogenetic predisposition.

In controls, the general state of health was recorded as self-perceived health and ruling out autoimmune, metabolic, cancer, or any repetitive disease or chronically treated. To establish the similarity in the exposure to possible triggers of the disease, both control individuals and patients belong to the same geographical area, which is expressed as latitude and longitude in the sociodemographic information Table 1.

**TABLE 1:** Clinical and sociodemographic information from SLE patients and controls.

Variable	SLE	Controls
Diagnosis Age, years, Mean ± SD	24. 5 ± 11.2	–
Age, years, Mean ± SD	39.7 ± 14.5	38 ± 15.3
Female	104 (89%)	120 (51%)
Male	13 (11%)	114 (49%)
Family	Grandparents and parents born at the same location	Grandparents and parents born at the same location
Latitude (Living locality)	19° 25′ N	19° 26′ N
Longitude (Living locality)	99° 7′ W	99° 8′ W
Urban/rural (Living locality)	Urban	Urban
Socioeconomic status	Low to middle	Low to middle
**Health status**		
Chronic diseases (Diabetes, hypertension)	6%	0%
SLEDAI % of individuals (score)	64% Inactive disease (0–2) 36% mild activity (3–4)	NA
SDI % of individuals (score)	84.6% (0–1) 13.67% (2–4) 1.73% (5–8)	NA
LupusQol % of individuals (score)	68% (>60)	NA
**Lupus phenotype and clinical manifestations**		
Articular	79.8%	
Serositis	29.7%	
Renal	65.6%	
Neurologic	16.4%	
Hematologic	64.8%	
Antiphospholipid syndrome	30.4%	
Other autoimmune diseases	34.5%	
ANAs	86%	
**Relatives’ information**		
Relative with SLE	18.6%	
Relative with autoimmune disease	29.4%	

*NA, not applicable; SLEDAI, SLE disease activity index; SDI, Systemic Lupus International Collaborating Clinics/American College of Rheumatology (SLICC/ACR) Damage Index; ANAs, antinuclear antibodies.*

Finally, the socioeconomic status was verified. The social worker’s department verified the patients’ socioeconomic strata using a validated instrument applied to all the institutions belonging to the Coordinating Commission of National Institutes and High Specialty Regional Hospitals (CCIHSHAE). This instrument includes variables with a numerical value to assign the socioeconomic level: monthly family income, occupation of the primary economic provider, monthly family expenses, housing, and family health status. The sum of variables gives an approximation of the socioeconomic level.

### Human Leukocyte Antigen Typing

#### Sanger Sequencing-Based Typing

Genomic DNA was obtained from whole blood using the QIAamp DNA mini kit (Qiagen, Valencia, CA, United States). DNA quality was assessed using a NanoDrop 2000 (Thermo Fisher Scientific, MA, United States) and Qubit Fluorometric Quantification (Invitrogen). The DNA integrity was evaluated by gel electrophoresis. Samples were stored at –20°C until analysis. The HLA typing was performed using a sequence-based method (SBM) described previously (Zúñiga et al., 2013).

Briefly, HLA class I typing was done by generic amplification of exons 2, 3, and 4 of each gene. For HLA class II, exon 2 and 3 of the HLA-DRB1 and -DQB1 genes were amplified using allele group-specific primer pairs. Polymerase chain reactions (PCRs) utilized 1.5 mm KCl, 1.5 mM MgCl2, 10 mM Tris-HCl (pH 8.3), 200 mM dNTPs, 10 pM of each primer, 30 ng of DNA, and 0.5 U of Taq DNA polymerase in a final volume of 25 μl. Amplifications were performed on a PE9700 thermal cycler (Applied Biosystems, Foster City, CA, United States) under the following cycling conditions: 95°C for 30 s, 65°C for 30 s, 72°C for 1 min, preceded by 5 min at 95°C and followed by a final elongation step at 72°C for 5 min. The amplified products were sequenced independently in both directions using BigDye^®^ Terminator v3.1 Cycle Sequencing Kit (Applied Biosystems^TM^) on the ABI PRISM^®^ 3500 Genetic Analyzer (Applied Biosystems^®^). Sequencing products were purified with the BigDye XTerminator^®^ Purification Kit (Applied Biosystems) to remove unincorporated BigDye^TM^ terminators and salts.

We analyzed data with matching allele assignment software (Applied Biosystems) using the IMGT/HLA sequence database alignment tool http://www.ebi.ac.uk/imgt/hla/align.html (Robinson, 2001). We solved ambiguities using group-specific sequencing primers (GSSPs) that had been previously validated (Lebedeva et al., 2011).

#### High-Resolution Typing by Next-Generation Sequencing

Next-generation sequencing Illumina^®^ TruSight^®^ HLA v2 Sequencing Panel (Illumina, San Diego, CA, United States) was also used to confirm HLA allele-level typing. We performed the process as the manufacturer recommends. Briefly, genomic DNA samples were adjusted to a working concentration of 10 ng/μL using Qubit equipment (Thermo Fisher Scientific, Waltham, MA, United States).

Generation of Long-range PCR templates. HLA-A,-B,-C,-DRB1, and -DQB1 loci were prepared using specific primers included in the TruSight HLA Pre 24 sample kit (Illumina) and MasterAmpTM Extra-Long DNA Polymerase (Lucien Corporation, Middleton, WI, United States).

Polymerase chain reactions were performed in a 96-well plate on the 9700 PE thermal cycler (Applied Biosystems/Thermo Fisher Scientific) using the following reagents proportions: 25 μl of HPM (HLA-PCR Mix), 2 μl of MasterAmpTM Extra-Long DNA Polymerase, 13 μl of water, and 5 μl of gDNA (10 ng/μl). Two PCR programs were performed for the fragment amplification of HLA loci. The first one for amplification of HLA-A, -B, -C, and -DRB1 loci, under the following conditions: initial denaturation at 94°C for 3 min, 30 cycles at 94°C for 30 s, 60°C for 2 min, 68°C for 15 min, 68°C for 10 min, and a final hold at 10°C.

The second PCR program for locus HLA-DQB1 was performed under the following conditions: 94°C for 3 min; followed by 10 cycles at 94°C for 30 s, 55°C for min, 72°C for 15 min; 20 cycles of 94°C for 30 s, 60°C for 2 min, 72°C for 15 min, 72°C for 10 min; and a final hold at 10°C. PCR products were confirmed by 1% agarose gel electrophoresis. The PCR clean-up was performed too.

Normalization and tagmentation. All loci PCR products’ concentrations were normalized using magnetic beads (LNA1, LNB1, TruSight HLA, Illumina). This process is accomplished for multiplex library preparation and sequencing. After normalization, 40 μl of each PCR product was used for fragmentation (800 and 1200 pb), and fragmentation buffers HTM and HTB (TruSight HLA Pre-PCR 24, Illumina) were added to the reaction (10 μl each) and incubated at 58°C for 12 min in the presence of sequencing primers. The purified fragmented PCR products were pooled, and adaptor addition was performed using the Nextera XT DNA sample preparation kit (Illumina). Amplification was performed based on the following PCR program: denaturation at 72°C for 3 min and 98°C for 30 s, followed by 10 cycles at 98°C for 10 s, 60°C for 30 s, 72°C for 5 min, and a final hold at 10°C. Appropriate clean-up was performed.

Sequencing. Seven microliters of the PCR sequencing products were denatured with 10 μl of 0.1N NaOH and sequenced on a MiSeq instrument using the paired-end 300 cycle (2 × 150 bp paired-end) MiSeq Reagent Kit (Illumina) following the manufacturer instructions.

#### Next-Generation Sequencing Data Analysis

After the sequencing, MiSeq reporter analysis software-generated FASTQ sequence files and BAM alignment files. Next, we generated allele calls using the Assign 2.0^TM^ TruSight HLA Analysis software. The software used reference sequences from the IMGT/HLA database (release 3.23.0.0).

### Statistical Analysis

#### Clinical and Sociodemographic Characteristics

We analyzed clinical and demographic variables with the IBM SPSS Statistics 26 program.

#### Human Leukocyte Antigen Class I and Class II Alleles and CEH Frequencies

Differences in HLA class I and II alleles and haplotypes frequencies between patients and controls were analyzed using X2, and *p*-Values less than 0.05 were considered statistically different. *P*-values were also corrected using the Bonferroni method (for allele frequencies, multiplying the original *p*-Value by the number of alleles). Odds ratios (OR) and 95% confidence intervals (95%CI) were calculated to measure association strength with the program Epi Info^TM^ 7.2 version (Centers for Disease Control and Prevention, 2011). We generated a Forest plot of HLA alleles and haplotypes, which showed statistical significance using R programming version 4.0.3 (R Core Team, 2020).

Hardy-Weinberg equilibrium (HWE) at a locus-by-locus level was calculated. HLA alleles data was faced with the hypothesis that the observed diploid genotypes are the product of a random union of gametes. To detect significant departure from HWE was followed an analogous Fisher’s exact test on a two-by-two contingency table but extended to a triangular contingency table of arbitrary size. The test was done using a modified version of the Markov-chain with the populations’ genetic computer program Arlequin version 3.5.2.2 (Guo and Thompson, 1992; Excoffier and Lischer, 2010).

Furthermore, we calculated the diversity values: observed heterozygosity (OH), expected heterozygosity (EH), and the polymorphic information content (PIC) for each locus (HLA-A, -C, -B, -DRB1, and -DQB1). CEH of known Mexican Native American, European, African, and Asian origin were assigned based on previously reported frequencies investigated as Most Probably Ancestry (MPA) in previous studies. MPA is based on the frequency occurrence of haplotypes (Cao et al., 2001; Yunis et al., 2003, 2005; Zúñiga et al., 2013). Individual alleles and haplotypes frequencies and locality of occurrence searching HLA tool can be found in Allele Frequency Net Database, the gold-standard data classification (Gonzalez-Galarza et al., 2020).

Linkage disequilibrium (LD) between HLA loci pairs was calculated based on delta (Δ), a LD coefficient, which measures the deviation from a random association between alleles of different loci. The results have been reported as relative delta (Δ′) values. Δ′ oscillates among values from -1 to 1. 1 represents the highest probability that a pair of alleles or DNA segments segregates as a block. In contrast, -1 represents the probability of a total random pairing (Excoffier and Lischer, 2010).

### Admixture Estimation

The maximum likelihood method was used to estimate SLE patients’ and healthy individuals’ admixture proportions using the population genetics LEADMIX software (Wang, 2003). Four major parental populations (*k* = 4) were evaluated, including a population per continental location according to the settlement history in Mexico. According to the availability of HLA data, the populations included were Mexican Native American, Southwestern European, Sub Saharan African, and Eastern Asian. HLA-B and -DRB1 were used as genetic estimators in the included populations. We estimated Mexican Native American contribution from Oaxaca Mixtecs data, a populace from southeastern Mexico (Arnaiz-Villena et al., 2014), and Chihuahua Tarahumaras, a northern Mexico population (García-Ortiz et al., 2006). Similarly, we estimated non-autochthonous parental populations’ contribution with data obtained from Southwestern European components from a representative sample from Spain (Catalunya, Navarra, Extremadura, Aragon, and Cantabria) (Enrich et al., 2019), Sub-Saharan African components from Zimbabwe Harare Shona Inhabitants (Louie, 2006b), and Eastern Asian components from the China Han populace (Trachtenberg et al., 2007).

### Genetic Distance and Principal Component Analysis

Genetic distances were assessed for the parental populations evaluated in the admixture estimation (*k* = 4) (Mexican Native American, Southwestern European, Sub-Saharan African, and Eastern Asian). This analysis was based on HLA-B. Besides, we included other Mexican Native American groups to deepen the comparison; the groups added were: Lacandon Mayans (Barquera et al., 2020f), Oaxaca Mixe (Hollenbach et al., 2001; Single et al., 2020), Oaxaca Zapotecans (Hollenbach et al., 2001), and Seri (Gorodezky, 2006). The genetic distance analysis included Nei’s distance performed with the Arlequin genetics population program. We generated graphics with an R programming extension added to Arlequin.

Principal Components Analysis (PCA) for fifty-six populations with HLA-B data available was performed using the BioVinci Software 2.0 to analyze the distribution of HLA-B alleles. There were included populations from three continental locations European, African, and Latin-American. The study groups SLE and controls HLA-B were adjusted to low-resolution. PCA included population data of African populations (7 populations): Burkina Faso Mossi (Modiano et al., 2001), Cameroon Yaounde (Pimtanothai et al., 2001), Ghana Ga-Adangbe (Norman et al., 2013), Kenya (Mack et al., 2006), Kenya Nandi (Cao et al., 2004), Uganda Kampala (Kijak et al., 2009), and Zimbabwe Harare Shona (Louie, 2006a). European populations (8 populations): England^∗^, France Lyon^∗^, Germany Essen^∗^, Ireland Northern (Williams et al., 1999; Middleton et al., 2000), Italy^∗^, Italy Sardinia (Grimaldi et al., 2001), Netherlands^∗^ and Spain (Enrich et al., 2019). Mexican Mestizo populations from Northern Mexico (7 populations): Baja California (Escobedo-Ruíz et al., 2020), Chihuahua (Pacheco-Ubaldo et al., 2020), Colima (Barquera et al., 2020b), Durango (González-Medina et al., 2020), Nuevo León (Barquera et al., 2020a), Sinaloa (Clayton et al., 2020), and Sonora (Uribe-Duarte et al., 2020). Mexican Mestizo populations from the Center of Mexico (14 populations): Aguascalientes (Bravo-Acevedo et al., 2020), Guanajuato (Pantoja-Torres et al., 2020), Guerrero (Juárez-Nicolás et al., 2020), Jalisco (Bravo-Acevedo et al., 2020), Mexico City Center, Mexico City Western, Mexico City Eastern, Mexico City Southern, Mexico City Northern (Barquera et al., 2020e), Michoacán (Ballesteros-Romero et al., 2020), Morelos (Ortega-Yáñez et al., 2020), Nayarit (Goné-Vázquez et al., 2020), Querétaro (Martínez-Álvarez et al., 2020), and San Luis Potosí (Hernández-Zaragoza et al., 2020). Mexican Mestizo populations from Southern Mexico (4 populations): Chiapas (Barquera et al., 2020d), Oaxaca (Hernández-Hernández et al., 2020), Quintana Roo (Medina-Escobedo et al., 2020) and Tabasco (Solís-Martínez et al., 2020). Native American Mexican populations (8 populations): Amerindian pooled population 1 (Tarahumaras, Mixtecs, and Zapotecan) (Hollenbach et al., 2001; García-Ortiz et al., 2006) and Amerindian pooled population 2 (Mixtecs and Tarahumaras), Mixe (Hollenbach et al., 2001), Mixtecs (Arnaiz-Villena et al., 2014), Nahuas (Vargas-Alarcón et al., 2003), Lacandon (Barquera et al., 2020f), Seri (Gorodezky, 2006), and Zapotecan (Hollenbach et al., 2001). Non-Mexican Latin-American populations (8 populations): Argentina Amerindian (Cerna et al., 1993), Bolivia Amerindian Aymara^∗^, Colombia^∗^, Costa Rica African-Caribbean (Arrieta-Bolaños et al., 2018), Costa Rica Amerindians (Arrieta-Bolaños et al., 2018), Ecuador^∗^, Nicaragua Managua^∗^, and Panama^∗^.

^∗^Populations from bone marrow or transplantation registry or the dataset is not associated with a publication or has not been yet published. The accessions are detailed in Data Availability Statement.

To establish the populations’ similarities and differences in the PCA plot, we generate a discrimination tree that shows high variance features (HLA alleles which frequency, distribution, and representativeness in populations are established as a criterion of discrimination, separation, or conjunction between the evaluated populations). The alleles which differentiate the populations better are showed in the discrimination tree. This is evaluated with the software algorithm base on the frequencies of each allele.

## Results

### Clinical and Sociodemographic Characteristics

The clinical and sociodemographic characteristics of the Mestizo Mexican SLE patients are summarized in Table 1. The SLE patients were 89% female, and they had a mean age of 39.7 ± 14.5 years. While controls were 51% female, and they had a mean age of 38.0 ± 15.0 years. Both groups were considered Mexican Mestizos. The latitude and longitude where the people live were very similar for both groups, took from The Allele Frequency Net Database (AFND). This data was important to validate that both groups are possible exposed to the same disease triggers in the same area. Both SLE and controls were cataloged as urban, which reports a similar utility. The lifestyle and habits could be another factor to restrict the exposition to the same triggers.

The socioeconomic status was defined with an instrument that allowed classifying SLE and Controls as low and middle socioeconomic strata individuals in similar proportions.

The general healthy state in the patients was corroborated by ACR classification, SLEDAI, and SDI questionaries with the clinical and history evaluation of the Rheumatologist. Different SLE phenotypes were found and are detailed in Table 1. Most of the patients showed over one clinical phenotype. The most common were arthritis (79.8%), renal (65.6%), and hematological (64.8%). The mean age of disease onset was 24.5 ± 11.2 years, and 34.5% of the patients presented a concomitant autoimmune disease. The most frequent were Graves’ disease and hypothyroidism. Other conditions less frequent were Sjogren’s syndrome, autoimmune acquired hemophilia, neuromyelitis optica, vitiligo, and scleroderma.

The healthy state of controls was verified by questionnaire. All participants expressed good self-perceived health: no diseases or chronic treatments and no impediment to daily activities.

### Genetic Diversity and Admixture

As expected, the HLA-B and HLA-DRB1 loci were the most polymorphic in both groups, whereas HLA-DQB1 was the least polymorphic locus. Diversity parameters, polymorphic information content, expected and observed heterozygosity values are shown in Table 2. Hardy-Weinberg Equilibrium analysis is displayed with the p corrected for each locus. HLA-DRB1 locus tends to have a marginal deviation from Hardy-Weinberg equilibrium after Bonferroni correction, which is being studied more deeply.

**TABLE 2:** Estimations of genetic diversity of HLA class I and class II loci in SLE patients and healthy Individuals.

HLA-locus	SLE				Healthy individuals			
	EH	OH	*p*C	PIC	EH	OH	*p*C	PIC
A	0.9060	0.9697	0.201	0.9002	0.8919	0.8718	0.531	0.9761
C	0.8922	0.9571	0.352	0.8879	0.8947	0.9009	0.196	0.9907
B	0.9654	0.9929	0.833	0.9608	0.9668	0.9487	0.216	0.9767
DRB1	0.9249	0.9489	0.080	0.9190	0.9193	0.9013	0.010	0.8350
DQB1	0.8610	0.9203	0.696	0.8600	0.8256	0.8205	0.269	0.9447

***pC**, p corrected value after Bonferroni correction; **OH**, observed heterozygosity; **EH**, expected heterozygosity; **PIC**, polymorphic information content.*

**FIGURE 1:**
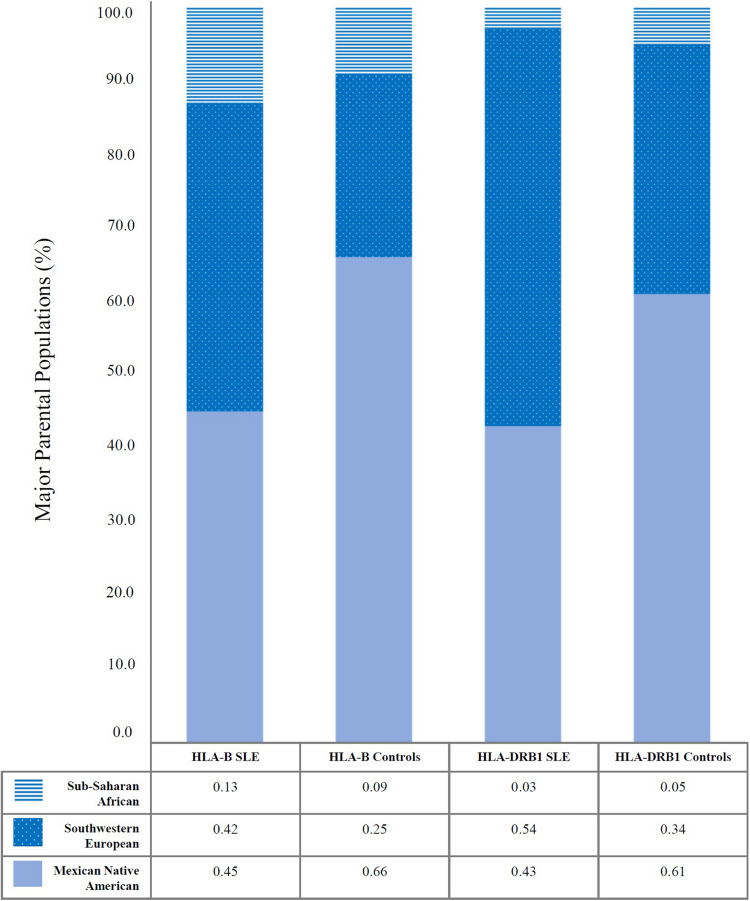
Ethnic admixture estimations revealed differences in Mexican Native American and Southwestern European components between Mexican SLE patients and healthy individuals. It was analyzed by Maximum likelihood approximation using HLA-B and HLA-DRB1. The values presented in Y-axis are the percentages of the main parental populations included (Mexican Native American, Southwestern European, and Sub-Saharan African). The table shows the relative frequencies from 0 to 1. Sub-Saharan African component is shown in blue horizontal lines, Southwestern European component is shown in blue with white points, and Mexican Native American component is shown in solid light blue. Eastern Asian ancestry was minimal, and it is not represented in the graphic.

Systemic lupus erythematosus patients have a higher non-autochthonous HLA gene load, while controls have a higher Mexican Native American HLA gene load. We performed the admixture estimations with the Maximum Likelihood method based on HLA-B and -DRB1 of four digits for each included population. HLA-B is the most polymorphic locus and, most of the time, is selected for admixture estimations. Besides, HLA-DRB1 is the locus most associated with autoimmunity. Both loci analyses conduct to the same conclusion: SLE patients have a higher proportion of Southwestern European ancestry, 48 ± 8% (Mean proportion between HLA-B and HLA-DRB1 ± Standard deviation) than healthy individuals, 30 ± 7%. Instead, SLE patients have a minor proportion of Mexican Native American ancestry, 44 ± 1% than healthy individuals 63 ± 4% and the Sub-Saharan African component appears to have a more similar distribution between SLE and controls 7 ± 7% and 6 ± 7%, respectively. The Asian admixture proportions are not showed; it was minimal. Detailed data for HLA-B and HLA-DRB1 are shown in Figure 1.

### Genetic Distances

**FIGURE 2:**
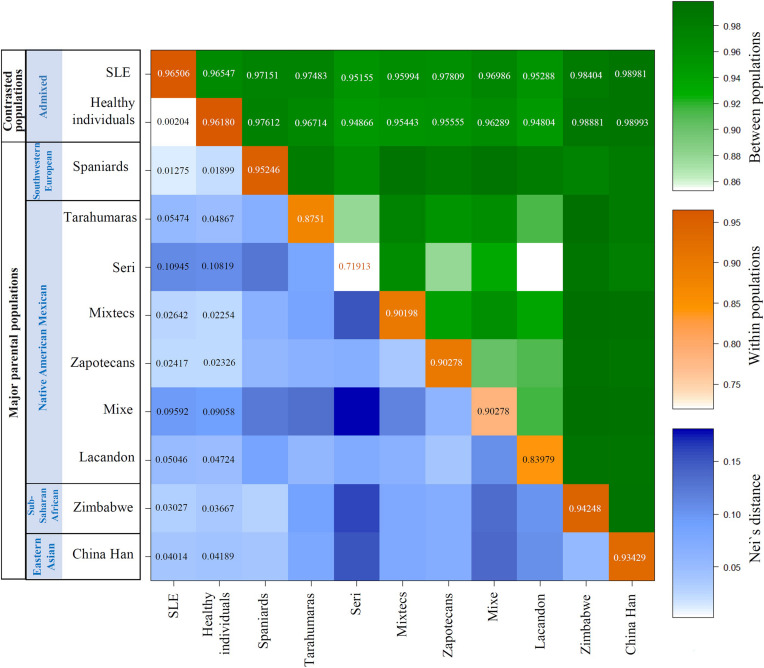
Genetic distances revealed differences in Mexican Native American and Southwestern European ancestry between the SLE and healthy individuals. In this matrix, we represent three different color scales, the average number of pairwise differences (π) between populations. Orange on diagonal: π within populations; Green above diagonal: πxy between pairs of populations Blue below diagonal: net number of nucleotide differences between populations (Nei’s distance). The interpretation is the same for the three scales; it is shown in the right color bars. To > value > genetic distance. To > intensity color > genetic distance.

As expected, because the admixed populations (SLE and healthy individuals) belong to the same region, the variations in genetic distances are low. However, the variations are consistent with the admixture analysis and showed statistical significance (*p*C < 0.05). Nei’s distances (d) have shown that SLE patients are closer to non-autochthonous populations (Southwestern European, Sub-Saharan African, and Eastern Asian) than healthy individuals, while healthy individuals are closer to autochthonous populations than the SLE group (Figure 2). Also, calculating the corrected average pairwise distance between populations gave the same information obtained through Nei’s distance. Additionally, the average number of pairwise differences within populations was considered to validate each populations’ genetic structure. As it is shown, the most isolated Mexican Native American groups (Seri, Lacandon, and Mixe, which are Mexican Native American groups that are geographically and culturally independent from other Mexican populations, implies a reduced or null gene flow from and to these populations, and consequently reduced diversity) showed fewer intra-group differences. These results are shown in a color matrix. The values corresponding to admixed groups (SLE and healthy) have been included in the matrix. The entire matrix values are shown in Supplementary Information 1 as population average pairwise differences (π). The same conclusions were reached with other genetic structure analysis as FST value, coancestry coefficients, and Slatkin linearized FSTs (Supplementary Figures 1–3).

**FIGURE 3:**
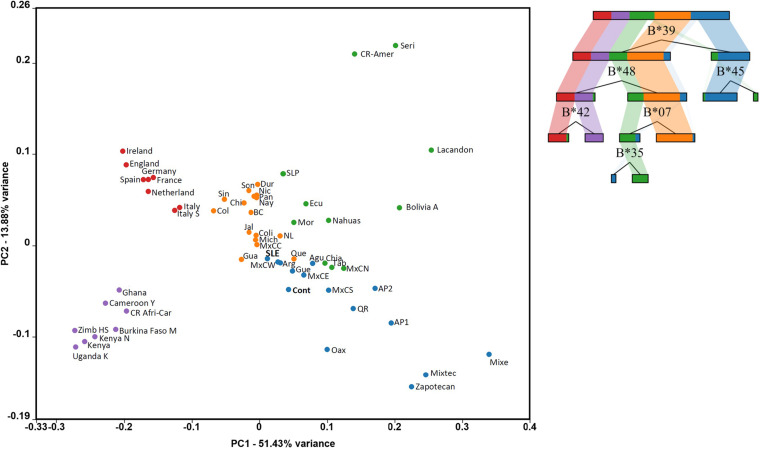
The principal component analysis (PCA) plot shows the SLE Mexico City particularities. Populations are colored by high variance features represented by the alleles in the decision tree shown on the right. Some populations match in color and continental location with the HLA allele frequency discrimination. Purple dots represent African, and red represent European. Orange, blue, and green dots represent Mestizo and Native populations from Mexico states and Central and South America, properly described in the “Materials and Methods” section. Burkina Faso M: Burkina Faso Mossi, Uganda K: Uganda Kampala, Cameroon Y: Cameroon Yaounde, Kenya N: Kenya Nandi, Ghana: Ghana Ga-Adangbe, Italy S: Sardinia, BC: Baja California, Coli: Colima, Sin: Sinaloa, Dur: Durango, Son: Sonora, and NL: Nuevo León, Nic: Nicaragua, Pan: Panama, Col: Colombia, Que: Queretaro, Gua: Guanajuato, Mich: Michoacán, MxCC: Mexico City Center. Agu: Aguascalientes, Gua: Guanajuato, Que: Queretaro, Gue: Guerrero, Mich: Michoacán, SLP: San Luis Potosí, Nay: Nayarit, Jal: Jalisco, MxCC: Mexico City Center, MxCW: Mexico City Western, MxCE: Mexico City Eastern, MxCS: Mexico City Southern, MxCN: Mexico City Northern. Chi: Chiapas, Tab: Tabasco, Oax: Oaxaca, QR: Quintana Roo. AP1: Amerindian pooled 1, AP2: Amerindian pooled 2, CR-Amer: Costa Rica Amerindian, CR Afri-Car: Costa Rica African-Caribbean. Ecu: Ecuador, Mor: Morelos, SLP: San Luis Potosí, Bolivia A: Bolivia Aymaras.

Further evidence of the differentiated distribution of immunogenetic diversity in Mexican mestizo groups (SLE and healthy individuals) can be seen in the principal component analysis (PCA) graph. Populations from three continental locations with HLA-B data were considered to construct the PCA. The objective was to visualize the differences between LES and Controls better. We opted to generate a visual discrimination tree, which calls the strongest elements that differentiate the included populations. The PCA point distribution is consistent with ethnic differences among the included populations. The separation of SLE and controls is consistent with the analysis of genetic distances. But it is observed that both the SLE groups and the controls are in the area of the plot where other groups of Mexican mestizos of Mexico City are found (MxCC, MxCW, MxCE, MxCS, MxCN, and MxCS; Centro Ciudad de México, West, East, South, and North, respectively). The distribution of the groups of Mexican mestizos in the PCA shows previous knowledge about Mexican mestizos. The northern states have higher European ancestry, while the southeast has more similarities among Mexican Native Americans (Figure 3).

Additionally, it can be observed how the Native American groups of Mexico present important differences with the mestizo populations. The genetics of Mexico recapitulates the substructure of the Native Americans and affects the biomedical traits. Therefore, although they seem distant and little related, these autochthonous populations confer complexity to the Mexican admixture. Thus, the immunogenetic diversity of the HLA system in Mexico correlates with the genetic structure of the underlying population (Figure 3).

HLA-A^∗^29:02∼C^∗^16:01∼B^∗^44^∗^03∼DRB1^∗^07:01∼DQB1^∗^02: 02 as a novel CEH of susceptibility identified in Mexican SLE patients.

The determination of allele and haplotype frequencies and the risk or protection conferred by the HLA alleles and haplotypes are shown in Tables 3–7 and Supplementary Tables 1–4. We found the frequency of a novel susceptibility haplotype incremented in SLE patients. The European block HLA-A^∗^29:02∼C^∗^16:01∼B^∗^44^∗^03∼DRB1^∗^07:01∼DQB1^∗^02:02 with *p*C = 0.02, OR = 6.7 (Table 7). None of the individual alleles that make up this CEH showed statistical significance in this study.

**TABLE 3:** Human leukocyte antigen-B allele frequencies in SLE patients and healthy individuals.

HLA-B alleles	SLE		Healthy individuals		*p*C	OR	95%IC	
	*N* = 143		*N* = 234					
	(286 alleles)		(468 alleles)					
					
	*n*	AF	*n*	AF				
B*08:01	**20**	**0.0699**	**3**	**0.0064**	**0.000003**	**11.7**	**3.43**	**39.59**
B*39:05	24	0.0839	37	0.0791	ns			
B*35:01	19	0.0664	27	0.0577	ns			
B*51:01	16	0.0559	28	0.0598	ns			
B*35:17	15	0.0524	18	0.0385	ns			
B*07:02	13	0.0455	19	0.0406	ns			
B*44:03	13	0.0455	13	0.0278	ns			
B*35:12	13	0.0455	18	0.0385	ns			
B*40:02	10	0.0350	25	0.0534	ns			
B*18:01	10	0.0350	8	0.0171	ns			
B*39:06	**8**	**0.0280**	**32**	**0.0684**	**0.03**	**0.4**	**0.18**	**0.86**
B*52:01	8	0.0280	10	0.0214	ns			
B*48:01	9	0.0315	20	0.0427	ns			
B*14:02	7	0.0245	15	0.0321	ns			
B*15:15	6	0.0210	15	0.0321	ns			
B*15:03	6	0.0210	2	0.0043	ns			
B*49:01	6	0.0210	9	0.0192	ns			
B*35:02	5	0.0175	2	0.0043	ns			
B*15:01	5	0.0175	10	0.0214	ns			
B*37:01	4	0.0140	4	0.0085	ns			
B*14:01	3	0.0105	4	0.0085	ns			
B*38:01	4	0.0140	6	0.0128	ns			
B*35:14	3	0.0105	7	0.0150	ns			
B*39:01	3	0.0105	5	0.0107	ns			
B*57:03	3	0.0105	1	0.0021	ns			
B*35:16	2	0.0070	3	0.0064	ns			
B*15:16	2	0.0070	1	0.0021	ns			
B*15:17	2	0.0070	3	0.0064	ns			
B*15:30	2	0.0070	8	0.0171	ns			
B*13:02	2	0.0070	6	0.0128	ns			
B*35:43	3	0.0105	9	0.0192	ns			
B*39:02	2	0.0070	10	0.0214	ns			
B*41:01	2	0.0070	5	0.0107	ns			
B*44:02	2	0.0070	5	0.0107	ns			
B*50:01	2	0.0070	4	0.0085	ns			
B*57:01	2	0.0070	7	0.0150	ns			
B*58:01	2	0.0070	3	0.0064	ns			
B*39:08	1	0.0035	3	0.0064	ns			
Other alleles	27							

***AF**, allele frequency; **ns**, not significant; **pC**, p corrected value using Bonferroni method; **OR**, odds ratio; **95%CI**, 95% confidence interval; **N**, total number of individuals; **n**, absolute frequency for each allele. Bold text highlights the results with statistical significance.*

**FIGURE 4:**
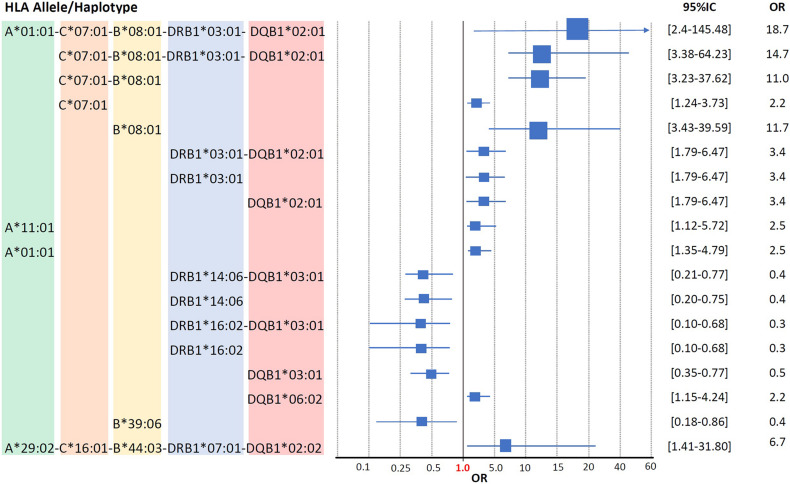
Human leukocyte antigen susceptibility and protection alleles and haplotypes in Mestizo Mexican SLE patients. The alleles and haplotypes, which showed statistically significant differences between SLE patients and healthy individuals (*p* < 0.05), are shown in the Forest plot. The Odds ratios and 95% interval confidence are included in the graphic. Blue boxes are not at scale.

The CEH HLA-A^∗^01:01∼C^∗^07:01∼B^∗^08:01∼DRB1^∗^03:01∼ DQB1^∗^02:01 is the highest risk factor to develop SLE in Mexicans mestizo patients from Mexico City. As shown in previous studies in low-resolution HLA typing (Granados et al., 1996; Vargas-Alarcón et al., 2001; Graham et al., 2007) and the current one in high-resolution HLA typing, the European conserved extended haplotype HLA-A^∗^01:01∼C^∗^07:01∼B^∗^08:01∼DRB1^∗^03:01∼DQB1^∗^02:01 (*p*C = 0.0004, OR = 18.7) has been found as the high-risk factor to develop SLE in Mexicans from Mexico City (Table 7). The individual loci statistical analysis has identified relative risks given by each of the alleles that compound this CEH (Figure 4). The relative risk observed is highly influenced by linkage disequilibrium with neighboring risk genes, both HLA and non-HLA (Supplementary Figure 4). Additionally, the allele HLA-A^∗^11:01 was found as a risk factor with no haplotype linkage, with *p*C = 0.035, OR = 2.5 (Figure 4 and Supplementary Table 1). This allele has previously identified in Malays and Chinese SLE patients (Mohd-Yusuf et al., 2011).

HLA-DRB1^∗^14:06∼DQB1^∗^03:01 and -DRB1^∗^16:02∼DQB1^∗^ 03:01 the protective Mexican Native American haplotypes which frequency is diminished in SLE patients from Mexico City, as it is shown in previous studies (Salgado-Galicia et al., 2020) and the current one. The HLA-DRB1^∗^14 has been previously found in Asians as -DRB1^∗^14:03 (Furukawa et al., 2014). The frequency of HLA class II protective alleles HLA-DRB1^∗^14:06 (*p*C = 0.006, OR = 0.4) and HLA-DRB1^∗^16:02 (*p*C = 0.006, OR = 0.3) were found diminished in the SLE group (Table 4). The Mexican Native American haplotypes, showed protection, the HLA-DRB1^∗^14:06∼DQB1^∗^03:01 (*p*C = 0.007, OR = 0.4) and HLA-DRB1^∗^16:02∼DQB1^∗^03:01 (*p*C = 0.006, OR = 0.3) (Tables 5, 6).

**TABLE 4:** Human leukocyte antigen-DRB1 allele frequencies in SLE patients and healthy individuals.

HLA-DRB1 alleles	SLE		Healthy individuals		*p*C	OR	95%IC	
	*N* = 143		*N* = 234					
	(286 alleles)		(468 alleles)					
					
	*n*	AF	*n*	AF				
DRB1*08:02	52	0.1818	91	0.1944	ns			
DRB1*04:07	27	0.0944	55	0.1175	ns			
DRB1*03:01	**29**	0.1014	**15**	0.0321	**0.0002**	**3.4**	**1.79**	**6.47**
DRB1*07:01	24	0.0839	33	0.0705	ns			
DRB1*15:01	20	0.0699	17	0.0363	ns			
DRB1*04:04	17	0.0594	31	0.0662	ns			
DRB1*11:04	10	0.0350	8	0.0171	ns			
DRB1*13:01	11	0.0385	12	0.0256	ns			
DRB1*14:06	**12**	0.0420	**47**	0.1004	**0.006**	**0.4**	**0.20**	**0.75**
DRB1*01:01	8	0.0280	9	0.0192	ns			
DRB1*04:11	7	0.0245	9	0.0192	ns			
DRB1*16:02	**5**	0.0175	**30**	0.0641	**0.006**	**0.3**	**0.10**	**0.68**
DRB1*04:01	3	0.0105	3	0.0064	ns			
DRB1*11:02	4	0.0140	4	0.0085	ns			
DRB1*13:03	5	0.0175	3	0.0064	ns			
DRB1*14:02	5	0.0175	11	0.0235	ns			
DRB1*04:05	3	0.0105	1	0.0021	ns			
DRB1*08:04	3	0.0105	2	0.0043	ns			
DRB1*15:03	4	0.0140	1	0.0021	ns			
DRB1*01:02	4	0.0140	11	0.0235	ns			
DRB1*01:03	3	0.0105	3	0.0064	ns			
DRB1*04:02	4	0.0140	10	0.0214	ns			
DRB1*08:01	2	0.0070	1	0.0021	ns			
DRB1*09:01	2	0.0070	1	0.0021	ns			
DRB1*12:01	1	0.0035	2	0.0043	ns			
DRB1*13:04	2	0.0070	1	0.0021	ns			
DRB1*04:03	1	0.0035	10	0.0214	ns			
DRB1*04:08	2	0.0070	1	0.0021	ns			
DRB1*04:10	1	0.0035	2	0.0043	ns			
DRB1*11:01	1	0.0035	6	0.0128	ns			
DRB1*13:02	3	0.0105	10	0.0214	ns			
DRB1*13:05	1	0.0035	1	0.0021	ns			
DRB1*16:01	1	0.0035	2	0.0043	ns			
DRB1*12:02	1	0.0035	3	0.0064	ns			
DRB1*14:01	1	0.0035	8	0.0171	ns			
Other alleles	7							

***AF**, allele frequency; **ns**, not significant; **pC**, p corrected value using Bonferroni method; **OR**, odds ratio; **95%CI**, 95% confidence interval**; N**, total number of individuals; **n,** absolute frequency for each allele. Bold text highlights the results with statistical significance.*

**TABLE 5:** Frequencies of HLA-DRB1∼DQB1 haplotypes in SLE patients and healthy individuals.

HLA-DRB1∼DQB1 Haplotypes	SLE *N* = 143			Healthy individuals			*p*C	OR	95%IC	
	(286 alleles)			(468 alleles)						
	*n*	HF	Δ′	*n*	HF	Δ′				
**African**										
DRB1*13:01∼DQB1*05:01	5	0.0175	0.410	1	0.0021	0.0159	ns			
DRB1*08:04∼DQB1*03:01	1	0.0035	0.216	2	0.0043	1.0000	ns			
DRB1*12:01∼DQB1*05:01	1	0.0035	1.000	1	0.0021	0.4632	ns			
**Amerindian**										
DRB1*08:02∼DRB1*04:02	52	0.1818	1.000	89	0.1902	0.9723	ns			
DRB1*04:07∼DQB1*03:02	27	0.0944	1.000	53	0.1132	0.9518	ns			
DRB1*14:06∼DQB1*03:01	**12**	**0.0420**	**1.000**	**46**	**0.0983**	**0.9717**	**0.007**	**0.4**	**0.21**	**0.77**
DRB1*16:02∼DQB1*03:01	**5**	**0.0175**	**1.000**	**30**	**0.0641**	**1.0000**	**0.006**	**0.3**	**0.10**	**0.68**
DRB1*14:02∼DQB1*03:01	4	0.0140	0.765	11	0.0235	1.0000	ns			
DRB1*04:11∼DQB1*04:02	1	0.0035	0.818	1	0.0021	–0.4595	ns			
**Asian**										
DRB1*11:02∼DQB1*03:01	2	0.0070	0.412	3	0.0064	0.6674	ns			
DRB1*09:01∼DQB1*03:03	1	0.0035	0.216	1	0.0021	1.0000	ns			
DRB1*13:02∼DQB1*05:01	1	0.0035	0.279	1	0.0021	0.0338	ns			
**Caucasian**										
DRB1*03:01∼DQB1*02:01	**29**	**0.1014**	**1.000**	**15**	**0.0321**	**1.0000**	**0.0002**	**3.4**	**1.79**	**6.47**
DRB1*15:01∼DQB1*06:02	18	0.0629	0.891	15	0.0321	0.8779	ns			
DRB1*11:04∼DQB1*03:01	8	0.0280	0.765	8	0.0171	0.0171	ns			
DRB1*13:01∼DQB1*06:03	6	0.0210	0.740	6	0.0128	1.0000	ns			
DRB1*04:01∼DQB1*03:02	2	0.0070	0.576	3	0.0064	1.0000	ns			
DRB1*07:01∼DQB1*03:03	2	0.0070	0.635	5	0.0107	0.4620	ns			
DRB1*11:01∼DQB1*03:01	1	0.0035	1.000	2	0.0043	0.1130	ns			
DRB1*04:02∼DQB1*03:02	4	0.0140	1.000	10	0.0214	1.0000	ns			
**Caucasian shared with other populations**										
DRB1*07:01∼DQB1*02:02	22	0.0769	0.908	28	0.0598	1.0000	ns			
DRB1*04:04∼DQB1*03:02	17	0.0594	1.000	29	0.0620	0.9144	ns			
DRB1*01:02∼DQB1*05:01	12	0.0420	1.000	11	0.0235	1.0000	ns			
DRB1*13:03∼DQB1*03:01	3	0.0105	0.529	3	0.0064	1.0000	ns			
DRB1*01:03∼DQB1*05:01	2	0.0070	0.640	3	0.0064	1.0000	ns			
DRB1*04:05∼DQB1*03:02	2	0.0070	0.576	1	0.0021	1.0000	ns			
DRB1*08:01∼DQB1*04:02	2	0.0070	1.000	1	0.0021	1.0000	ns			
DRB1*11:02∼DQB1*03:19	2	0.0070	0.493	1	0.0021	0.2419	ns			
DRB1*04:03∼DQB1*03:02	1	0.0035	1.000	10	0.0214	1.0000	ns			
DRB1*12:02∼DQB1*03:01	1	0.0035	1.000	3	0.0064	1.0000	ns			
DRB1*13:02∼DQB1*06:04	1	0.0035	1.000	9	0.0192	0.8978	ns			
DRB1*13:05∼DQB1*03:01	1	0.0035	1.000	1	0.0021	1.0000	ns			
DRB1*14:01∼DQB1*05:03	1	0.0035	1.000	8	0.0171	1.0000	ns			
DRB1*16:01∼DQB1*05:02	1	0.0035	1.000	1	0.0021	0.4968	ns			

***HF**, haplotype frequency; ns, not significant; **pC**, p corrected value using Bonferroni method; **OR**, odds ratio; **95%CI**, 95% confidence interval. **N**, total number of individuals**, n**, absolute frequency for each allele; Δ′, linkage disequilibrium value. Bold text highlights the results with statistical significance.*

**TABLE 6:** Frequencies of HLA-C∼B∼DRB1∼DQB1 haplotypes in SLE patients and healthy individuals.

HLA-C∼B∼DRB1∼DQB1 Haplotypes	SLE *N* = 143			Healthy controls			*p*C	OR	%95IC	
	(286 alleles)			*N* = 234 (468 alleles)						
	*n*	HF	Δ′	*n*	HF	Δ′				
**Amerindian**										
C*07:02∼B*39:05∼DRB1*04:07∼DQB1*03:02	16	0.0559	0.663	19	0.0406	0.502	ns			
C*04:01∼B*35:12∼DRB1*08:02∼DQB1*04:02	10	0.0350	0.717	7	0.0150	0.305	ns			
C*04:01∼B*35:17∼DRB1*08:02∼DQB1*04:02	10	0.0350	0.591	14	0.0299	0.726	ns			
C*01:02∼B*15:15∼DRB1*08:02∼DQB1*04:02	5	0.0175	0.795	8	0.0171	0.525	ns			
C*03:03∼B*52:01∼DRB1*14:06∼DQB1*03:01	5	0.0175	0.826	3	0.0064	0.446	ns			
C*07:02∼B*39:06∼DRB1*14:06∼DQB1*03:01	4	0.0140	0.478	16	0.0342	0.548	ns			
C*03:04∼B*40:02∼DRB1*08:02∼DQB1*04:02	3	0.0105	0.181	2	0.0043	–0.044	ns			
C*01:02∼B*15:30∼DRB1*08:02∼DQB1*04:02	2	0.0070	1.000	4	0.0085	0.383	ns			
C*08:01∼B*48:01∼DRB1*08:02∼DQB1*04:02	2	0.0070	1.000	8	0.0171	1.000	ns			
C*01:02∼B*15:01∼DRB1*16:02∼DQB1*03:01	1	0.0035	0.321	2	0.0043	0.237	ns			
C*01:02∼B*35:43∼DRB1*08:02∼DQB1*04:02	1	0.0035	0.181	2	0.0043	0.040	ns			
C*07:02∼B*39:01∼DRB1*08:02∼DQB1*04:02	1	0.0035	0.181	2	0.0043	0.383	ns			
C*15:02∼B*51:01∼DRB1*08:02∼DQB1*04:02	1	0.0035	1.000	4	0.0085	0.314	ns			
**Caucasian**										
C*07:01∼B*08:01∼DRB1*03:01∼DQB1*02:01	**17**	**0.0594**	**0.883**	**2**	**0.0043**	**1.000**	**0.00001**	**14.7**	**3.38**	**64.23**
C*16:01∼B*44:03∼DRB1*07:01∼DQB1*02:02	9	0.0315	0.891	6	0.0128	0.734	ns			
C*07:02∼B*07:02∼DRB1*15:01∼DQB1*06:02	6	0.0210	0.514	7	0.0150	0.448	ns			
C*05:01∼B*18:01∼DRB1*03:01∼DQB1*02:01	4	0.0140	1.000	3	0.0064	0.587	ns			
C*08:02∼B*14:02∼DRB1*01:02∼DQB1*05:01	4	0.0140	1.000	5	0.0107	0.441	ns			
C*05:01∼B*44:02∼DRB1*04:02∼DQB1*03:02	2	0.0070	1.000	2	0.0043	0.489	ns			
C*06:02∼B*13:02∼DRB1*07:01∼DQB1*02:02	2	0.0070	1.000	4	0.0085	0.787	ns			
C*07:01∼B*57:01∼DRB1*07:01∼DQB1*03:03	1	0.0035	1.000	3	0.0064	1.000	ns			
**Caucasian shared with other populations**										
C*04:01∼B*35:01∼DRB1*04:04∼DQB1*03:02	2	0.0070	0.077	3	0.0064	0.147	ns			
C*03:03∼B*52:01∼DRB1*08:02∼DQB1*04:02	1	0.0035	∼0.103	2	0.0043	0.176	ns			
C*06:02∼B*50:01∼DRB1*03:01∼DQB1*02:01	1	0.0035	0.442	2	0.0043	0.483	ns			
C*08:02∼B*14:01∼DRB1*07:01∼DQB1*02:02	1	0.0035	0.276	3	0.0064	0.734	ns			
C*08:02∼B*14:02∼DRB1*03:01∼DQB1*02:01	1	0.0035	0.044	2	0.0043	0.155	ns			
**Unknown**										
C*04:01∼B*35:01∼DRB1*08:02∼DQB1*04:02	4	0.0140	0.099	3	0.0064	0.012	ns			
C*07:02∼B*39:05∼DRB1*08:02∼DQB1*04:02	4	0.0140	∼0.064	5	0.0107	–0.227	ns			
C*08:01∼B*48:01∼DRB1*04:04∼DQB1*03:02	3	0.0105	0.335	3	0.0064	0.147	ns			
C*04:01∼B*35:14∼DRB1*16:02∼DQB1*03:01	2	0.0070	0.661	4	0.0085	0.644	ns			
C*07:02∼B*39:06∼DRB1*04:07∼DQB1*03:02	2	0.0070	0.170	4	0.0085	0.039	ns			
C*01:02∼B*15:01∼DRB1*08:02∼DQB1*04:02	1	0.0035	0.181	3	0.0064	0.294	ns			
C*06:02∼B*37:01∼DRB1*01:03∼DQB1*05:01	1	0.0035	0.493	2	0.0043	0.665	ns			

*HF, haplotype frequency; ns, not significant; pC, p corrected value using Bonferroni method; OR, odds ratio; 95%CI, 95% confidence interval. N, total number of individuals, n, absolute frequency for each allele; Δ′, Linkage disequilibrium value. Bold text highlights the results with statistical significance.*

**TABLE 7:** Frequencies of HLA∼A/∼C/∼B/∼DRB1/∼DQB1 haplotypes in SLE patients and healthy individuals.

HLA∼A/∼C/∼B/∼DRB1/∼DQB1 Haplotypes	SLE			Healthy Individuals			*p*C	OR	95%IC	
	*N* = 286			*N* = 468						
	*n*	HF	Δ′	*n*	HF	Δ′				
**Amerindian**										
A*02:01∼C*04:01∼B*35:12∼DRB1*08:02∼DQB1*04:02	6	0.0210	0.493	4	0.0085	0.444	ns			
A*68:03∼C*07:02∼B*39:05∼DRB1*04:07∼DQB1*03:02	5	0.0175	0.380	5	0.0107	0.283	ns			
A*02:06∼C*07:02∼B*39:05∼DRB1*04:07∼DQB1*03:02	4	0.0140	0.203	5	0.0107	0.185	ns			
A*02:01∼C*01:02∼B*15:15∼DRB1*08:02∼DQB1*04:02	3	0.0105	0.239	3	0.0064	0.190	ns			
A*02:01∼C*04:01∼B*35:17∼DRB1*08:02∼DQB1*04:02	2	0.0070	0.013	7	0.0150	0.352	ns			
A*02:06∼C*04:01∼B*35:17∼DRB1*08:02∼DQB1*04:02	2	0.0070	0.174	2	0.0043	0.517	ns			
A*24:02∼C*04:01∼B*35:12∼DRB1*08:02∼DQB1*04:02	2	0.0070	0.059	2	0.0043	0.141	ns			
A*24:02∼C*07:02∼B*39:06∼DRB1*14:06∼DQB1*03:01	2	0.0070	0.412	12	0.0256	0.699	ns			
A*31:01∼C*04:01∼B*35:17∼DRB1*08:02∼DQB1*04:02	2	0.0070	0.161	2	0.0043	0.069	ns			
A*68:01∼C*01:02∼B*15:15∼DRB1*08:02∼DQB1*04:02	2	0.0070	0.368	3	0.0064	0.321	ns			
A*02:01∼C*08:01∼B*48:01∼DRB1*08:02∼DQB1*04:02	1	0.0035	0.366	3	0.0064	0.190	ns			
**Caucasian**										
A*01:01∼C*07:01∼B*08:01∼DRB1*03:01∼DQB1*02:01	**11**	**0.0385**	**0.656**	**1**	**0.0021**	**0.481**	**0.0004**	**18.7**	**2.40**	**145.48**
A*29:02∼C*16:01∼B*44:03∼DRB1*07:01∼DQB1*02:02	**8**	**0.0280**	**0.884**	**2**	**0.0043**	**0.316**	**0.02**	**6.7**	**1.41**	**31.80**
A*02:01∼C*07:02∼B*07:02∼DRB1*15:01∼DQB1*06:02	3	0.0105	0.366	4	0.0085	0.444	ns			
A*30:02∼C*05:01∼B*18:01∼DRB1*03:01∼DQB1*02:01	3	0.0105	0.742	3	0.0064	1.000	ns			
A*02:01∼C*16:01∼B*44:03∼DRB1*07:01∼DQB1*02:02	1	0.0035	–0.475	2	0.0043	0.135	ns			
**Unknown**										
A*02:01∼C*03:03∼B*52:01∼DRB1*14:06∼DQB1*03:01	4	0.0140	0.746	2	0.0043	0.568	ns			
A*02:01∼C*07:02∼B*39:05∼DRB1*04:07∼DQB1*03:02	3	0.0105	–0.114	6	0.0128	0.113	ns			
A*02:01∼C*01:02∼B*15:30∼DRB1*08:02∼DQB1*04:02	2	0.0070	1.000	3	0.0064	0.676	ns			
A*02:01∼C*01:02∼B*35:43∼DRB1*08:02∼DQB1*04:02	1	0.0035	1.000	2	0.0043	1.000	ns			
A*02:01∼C*04:01∼B*35:01∼DRB1*08:02∼DQB1*04:02	1	0.0035	0.049	2	0.0043	0.568	ns			
A*24:02∼C*07:02∼B*39:06∼DRB1*04:07∼DQB1*03:02	1	0.0035	0.412	2	0.0043	0.399	ns			
A*30:01∼C*06:02∼B*13:02∼DRB1*07:01∼DQB1*02:02	1	0.0035	0.494	4	0.0085	1.000	ns			
A*68:01∼C*07:02∼B*39:01∼DRB1*08:02∼DQB1*04:02	1	0.0035	1.000	2	0.0043	1.000	ns			

*HF, haplotype frequency; ns, no significant; pC, p corrected value using Bonferroni method; OR, odds ratio; 95%IC, 95% interval confidence; N, total number of individuals; n, absolute frequency for each allele; Δ′, Linkage disequilibrium value. Bold text highlights the results with statistical significance.*

## Discussion

The HLA sequencing has led both to identify a new susceptibility block HLA-A^∗^29:02∼C^∗^16:01∼B^∗^44^∗^03∼DRB1^∗^ 07:01∼DQB1^∗^02:02 and to determine the admixture print which distinguishes patients and healthy individuals in Mexico City. Therefore, alleles and haplotypes of susceptibility and protection found, both the previously described and the new one, will be analyzed, emphasizing discussing the influence of ethnic admixture and the genetic load of parental populations in the development of lupus.

The CEH associated for the first time with SLE development in Mexicans was the HLA-A^∗^29:02∼C^∗^16:01∼ B^∗^44^∗^03∼DRB1^∗^07:01∼DQB1^∗^02:02. This CEH has been cataloged in previous studies as European (Szilágyi et al., 2010). Only the isolated allele HLA-DRB1^∗^07 has been previously associated with antiphospholipid syndrome in SLE patients from Mexico City (Granados et al., 1997). However, this CEH has been a risk factor in Mexican patients diagnosed with Achalasia (Furuzawa-Carballeda et al., 2018). This condition is a motility disorder of the esophagus with abnormalities in the neurons that controls peristaltic movements, whose underlying cause is unknown. Notably, previous studies conducted in other populations suggested that achalasia patients have increased frequency of HLA-DRB1^∗^15 and -DQB1(eight-amino-acid insertion in the cytoplasmic tail of HLA-DQβ1) alleles in an ethnicity-specific manner. This fact proposes an immunogenetic mechanism (Verne et al., 1999; Gockel et al., 2014; Becker et al., 2016). However, in this study, no SLE patient who carries this CEH showed some achalasia symptomatology. Probably, the immunosuppressive treatment might mask achalasia symptoms or prevents the development in predisposed individuals. A congruent reason could be that the mean age of achalasia onset in Mexicans (42.3 ± 15.8 years) is slightly superior to SLE (26.5 ± 12.2 years). The data needs further research because the shared susceptibility conferred by this CEH could mean a share immunopathological mechanism depending on ethnic background. The onset of either lupus or achalasia could rely on the triggers, which might differ for each condition.

Achalasia onset has been associated with varicella herpes zoster virus previous infection (Becker et al., 2016). In contrast, SLE development has been associated with other viruses’ infections as Epstein-Barr virus (EBV), parvovirus B19 (B19V), and human endogenous retroviruses (HERVs) (Quaglia et al., 2021). Interestingly, the novel haplotype associated with SLE in Mestizo Mexicans from Mexico City is in 2.8% of these individuals, while only in 0.43% of healthy Mexicans, 3.42% in Spaniards (Enrich et al., 2019), and surprisingly in 3.84% of Achalasia Mexican patients (Furuzawa-Carballeda et al., 2018). Therefore, the increased frequency of this new haplotype in Mexican Mestizo patients may represent the convenience of preserving this haplotype as an immunological advantage against pathogens.

On the other hand, it is observable that the percentages of this haplotype in SLE and Achalasia Mexicans are similar to healthy Spaniards. So, we would expect scenarios with similar prevalence and disease phenotypes if it only depended on HLA susceptibility alleles. Unfortunately, there are no official statistics about the prevalence and incidence of SLE in Mexico. Still, reports of SLE in Hispanics show high SLE prevalence in Hispanics than Spaniards (138/100000 and 17.5 to 34.1/100000 per inhabitants per year, respectively) (Atisha-Fregoso et al., 2011; Izmirly et al., 2017; Rees et al., 2017). Furthermore, some severe manifestations such as kidney damage are also more evident in Hispanics (62%) than Caucasian patients (25%). In addition, activity disease score as Systemic Lupus Activity Measure revised (SLAM-R) has been found highest in Hispanic than Caucasian patients (Fernández et al., 2007). Thus, all the above shows that the severity and clinical manifestations differ in an ethnic-dependent manner even when susceptibility haplotypes like the one found in this study are shared between populations. Therefore, the importance of population genetic studies and admixture estimations on Countries with high variability as Mexico is. No less important, there is a conjunction of variables as the availability and access to medical services and treatments that modify the severity phenotype in different populations. This factor always is important to consider before concluding about ethnicity and admixture disparities among populations.

The ethnic background and admixture influence the development of SLE and autoimmunity in Mexicans. This assumption was introduced because the haplotype HLA-B8-DR3 is found in high frequencies in the Caucasian population and has a considerable prevalence of SLE and other autoimmune diseases (Awdeh et al., 1983; Alper et al., 1986; Szilágyi et al., 2010). In contrast, the Mexican Native American populations lack both the HLA-B8-DR3 haplotype and SLE cases. Therefore, the high frequency of HLA-B8-DR3 in SLE Mexican patients from Mexico City has been explained as the product of the introduced alleles and haplotypes during the Spaniard’s arrival to the Americas. Surprisingly, SLE is more prevalent and aggressive in Mexicans than Europeans. Since the origin of the susceptibility alleles is from Europeans, it would be expected that the severity of the disease and the phenotypes would be similar. However, many elements can intervene to generate a unique scenario in Mexico. For example, the specific interaction of Amerindian, European, and other populations genes influenced by the architecture and tridimensional arrangement of the genes in each mixed population. Not least, the pressure exerted by local triggers and the environment itself.

The complex scenario of autoimmunity in Mexico is framed in that the susceptibility varies as the ethnic admixture pattern does. It has been corroborated in SLE studies. For example, SLE Mestizo patients in Guadalajara (northwestern Mexico) have the haplotype HLA-DRB1^∗^15-DQA1^∗^01:02-DQB1^∗^06:02 as a susceptibility factor (Cortes et al., 2004) while SLE Mestizo patients from Tapachula, Chiapas (southeast of Mexico) have the susceptibility alleles HLA-DRB1^∗^15 and -DRB1^∗^16 (Sepúlveda Delgado et al., 2018). But neither in Guadalajara nor in Tapachula, the HLA-DRB1^∗^03:01 is a susceptibility factor. However, HLA-DRB1^∗^03:01 is one of the main susceptibility alleles found in this study and other previously conducted in Mexico City (Granados et al., 1996; Vargas-Alarcón et al., 2001). Parallel to these susceptibility differences, independent studies of population genetics have demonstrated de admixture variability among Guadalajara, Tapachula, and Mexico City (current study). The Native American Mexican load in Guadalajara, Tapachula, and Mexico City is ∼44%, ∼72%, ∼63%, respectively, while the European is ∼48%, ∼26%, ∼30%, and the African is ∼8%, ∼2%, ∼6% (Barquera et al., 2020d; Bravo-Acevedo et al., 2020). Therefore, it looks like admixture proportions in each Mexican State or region could drive toward specific susceptibilities for the same disease. However, it is not only the variation of admixture across Mexico. The presence of different triggers in each region could shape a more comprehensive scenery.

Therefore, the influence of admixture variations in SLE susceptibility could be briefly explained as follows: having a more European HLA load increases the chances of carrying a risk haplotype of this origin. Likewise, there are risk alleles and haplotypes of African and Asian origin; however, because the percentage of these ancestries is low in Mexican Mestizos, they are less likely to carry them. HLA alleles have been conserved in the Mexican Mestizo, probably because they represent an immunological advantage against infections by common pathogens in Mexico. However, the cost of efficiency in pathogen clearance has predisposed to immune hyperresponsiveness, prolonging inflammatory pathways, and leading to autoimmunity. Also, the advance in medicine and better treatments have augmented the survival, life expectancy, and quality of life, which allows the preservation of susceptibility alleles. All the above might be part of the eventual increase in statistics of autoimmune diseases in Mexico, a recent phenomenon caused mainly due to better diagnosis, but influenced by the recently acquired HLAs that have contributed to the fitness of Mexican Mestizos against local pathogens, but with less tolerance to the presence of triggers. This means the carriers of susceptibility alleles or haplotypes are “selected” to manifest the disease based on the presence of the triggers. Therefore, this selection includes individuals with susceptibility alleles and, since those with higher European ancestry are those that most likely have susceptibility alleles; these are the individuals that enrich the autoimmune group.

In truth, there are many assumptions for which further investigation should be carried out. However, the above assumptions could explain why the SLE group differs in admixture proportions compared to control individuals. In studies like the current, it is necessary to ensure the comparability of the groups of patients and controls since useful and accurate results depend on that data quality. Recently published characterization of individuals from Mexico City has been an additional resource to validate the admixture estimations in our control group. In this study were studied 1217 individuals northern (*N* = 751), southern (*N* = 52), eastern (*N* = 79), western (*N* = 33), and central (*N* = 152) Mexico City and surroundings. Admixture estimates are very similar to the calculations performed by Maximum likelihood in our study, being Native American Mexican 63.85%, European 28.53%, and African 7.61% (Barquera et al., 2020e).

Further evidence is shown in the PCA plot; our Mexican admixed samples (SLE and Healthy individuals) are separated from the non-autochthonous populations, which was expected. But, notably, the “Mestizo” sample of SLE patients from Mexico City is not overlapped with Controls.

Systemic lupus erythematosus is closer to non-autochthonous populations, while Healthy individuals are closer to autochthonous populations, congruent with admixture estimations and genetic distances. However, SLE patients retain their identity with the States of Central Mexico, which is expected because SLE patients belong to Mexico City; but, the percentages of ancestry differ, having a greater European load than expected for individuals from Mexico City.

This fact is particularly striking because we are talking about Mestizo Mexican Individuals who share the locality of born and the demographic history of settlement (colonial period) but have notable differences in ancestry proportions. This ambivalence in the SLE group could be explained by the fact that the local Native American alleles that confer identity to the Central zone of Mexico are conserved (which is detected in the PCA). At the same time, the advantageous foreign alleles have also been conserved, slightly modifying the proportions of the ancestry of the patients (admixture estimations and genetic distances) and augmenting the possibilities of carrying susceptibility foreign HLA alleles.

The ancestry variation in individuals with SLE, as said before, could mean that some non-autochthonous HLA alleles have been conserved because these alleles represent an immunogenetic advantage, as demonstrated for HLA-DRB1^∗^03. For instance, some naturally processed HLA-DR3-restricted Human herpesvirus 6B (HHV-6B) peptides are recognized broadly with polyfunctional and cytotoxic CD4 T-cell responses (Becerra-Artiles et al., 2019). However, this convenient immunogenetic mechanism for clearing infections might be the detonator for immunological hyperreactivity, which marks the onset of the disease. Viruses infections associated with SLE have been described previously and are ethnically associated with SLE pathogenesis, specific HLA class II alleles, and the development of antinuclear antibodies, the hallmark of SLE have been associated in the same studies (Perl et al., 1995; Magistrelli et al., 1999; Quaglia et al., 2021).

Likewise, Nei’s distance data is congruent with the information given by both the susceptibility and protection haplotypes and the admixture analysis. It was found that the SLE group is closer to foreign parental populations (Southwestern European, Sub-Saharan African, and Eastern Asian) than the healthy individuals. In contrast, healthy individuals are nearer to Mexican Native American populations evaluated (Mixtecs, Zapotecans, Tarahumaras, Lacandon, Mixe, and Seri). As expected for intra-specie analysis for the same geographic area, the genetic distance variability is low, but the differences are consistent and significant. Corrected average pairwise differences gave the same information.

The matrix of genetic distances reflects the differences between the Mestizos of Mexico City (SLE and controls), Figure 2. As mentioned before about PCA, SLE and controls are Mestizos from the same geographical area, so it would be expected that there would be no variation in admixture proportions. But indeed, there is. The matrix shows consistent variation indicating SLE patients are genetically more similar to non-autochthonous populations. In comparison, healthy individuals are genetically more similar to Mexican Native American populations. However, some aspects about populations included are worth exploring to substantiate the validity of the comparison.

Regarding the notable genetic variation of the Mexican indigenous groups included, it was expected. The presented Fst matrix and values (Supplementary Figure 1), and the other calculations as coancestry and Slatkin’s Fst (Supplementary Figures 2, 3), reflect a bit of what has been described for the native populations that inhabited Mexico. It has been corroborated a striking genetic stratification among indigenous populations within Mexico at varying degrees of geographic isolation. Some groups are differentiated as Europeans are from East Asians. Seris and Lacandons are good examples (Infante et al., 1999; Barquera et al., 2020c); these groups are exposed to high levels of genetic drift and isolation. However, the value of including them as Mexican Native American parental populations is that present-day Mexicans’ genetic composition recapitulates ancient Native American substructure, despite the potential homogenizing effect of postcolonial admixture. Fine-scale population structure going back centuries is not merely a property of isolated or rural indigenous communities. Cosmopolitan populations still reflect the underlying genetic ancestry of local native populations, arguing for a strong relationship between the indigenous and the Mexican mestizo population, albeit without the extreme drift exhibited in some current indigenous groups (Moreno-Estrada et al., 2014).

Another notable feature is the genetic distances between the selected non-indigenous populations. Mainly, the Fst indicates an unexpected but explainable closeness between Zimbabwe and the Spanish. The characteristics of the Zimbabwe Harare Shona sample and previous periods of European colonization may have had a specific influence on this population that we cannot trace but hypothesize. Non-local HLA alleles may have been conserved as an immunological advantage, which has already been mentioned for Mexican Mestizos, additional to the MHC Class III genes with immunological functions conserved due to the linkage disequilibrium with the possibly acquired HLA Class I and Class II variants. Therefore, it should be noted that although the European load is likely to be low in this group of sub-Saharan Africans, the variants that contribute to fitness can be conserved and detected in the genetic distance calculations.

Furthermore, estimates of admixture by HLA are robust. In other studies, in Mexico City individuals, the admixture estimates are similar to the current study. It should be noted that these studies used HLA parental populations data different from that used in this study. Estimates with other techniques such as STRs have also generated similar results (Juárez-Cedillo et al., 2008). Thus, if the selected parental populations agree with the settlement background in Mexico, they can bring us the correct information about admixture and the genetic distances.

Finally, the CEH described as a susceptibility factor for Europeans the HLA-A^∗^01:01∼C^∗^07:01∼B^∗^08:01∼DRB1^∗^02:01 ∼DQB1^∗^02:01 has been found as a high-risk factor for developing SLE. This CEH has a powerful influence on SLE development and other autoimmune diseases in mestizo Mexican individuals from Mexico City (Granados et al., 1996) and other populations. At first glance, this haplotype’s relative risk appears to have a summative effect by the alleles that make up it; as more HLA alleles are added to the haplotype, the risk increases. However, the relative risk conferred by HLA-B^∗^08:01, HLA-DRB1^∗^03:01, and haplotypes containing them confirm that the risk is not summative but conferred by the genes in linkage disequilibrium with these variants. Besides, this linkage has been turned more complicated during the HLA research course. Early in the study of HLA was described a strong linkage disequilibrium not only among HLA genes of the haplotype HLA-B8-DR3 but between HLA and complement (C2, C4a, C4b, and Factor B) (Black et al., 1982; Wescott et al., 1987; Picceli et al., 2016). Currently, it is known that a considerable number of variants, mainly of MHC class three, are in linkage disequilibrium with the variants of B and DR indicated above, which increases the genetic risk load of the individual with lupus.

Models have been developed to confirm the susceptibility conferred by genes in the MHC III region (non-classical HLA and non-HLA genes) linked to HLA-B and -DRB1. The most important linked genes using HLA-DRB1^∗^03:01 as covariant are: the proto-oncogene Notch homologue 4 (NOTCH4), the MHC class I polypeptide–related sequence A and B (MIC-A and MIC-B), the steroid 21-hydroxylase (CYP21A2), (Partanen et al., 1988; Morris et al., 2012) the three 70 kDa heat-shock proteins (HSPA1A, HSPA1B, HSPA1L), (Mišunová et al., 2017), natural cytotoxicity triggering receptor 3 (NCR3); nuclear factor kappa light chain gene enhancer in B cells inhibitor-like 1 (NFKBIL1), allograft inflammatory factor 1 (AIF1) (Dorak et al., 2006); and the three Tumor Necrosis Factor genes (Lymphotoxin-beta, Lymphotoxin-alpha, and TNF-α) (Zúñiga et al., 2001; Ramírez-Bello et al., 2018). Important variants of the genes mentioned above have been deeply studied in Caucasians, and linkage disequilibrium with HLA-B^∗^08:01 and -DRB1^∗^03:01 has been confirmed (Dorak et al., 2006). This type of study is a prospect to be carried out in the Mexican population to determine the integrity of the MHC blocks conserved in Mexican Mestizos and how the variants could have been fixed or discarded, giving the specific characteristics of SLE in Mexicans.

## Conclusion

In conclusion, the genetic background and the Mexican admixture heterogeneity define part of the dynamic of SLE in Mestizo Mexican patients. Considering the regional distribution of genetic susceptibility and the ethnic barriers can be a useful biomedical tool. Increasingly, the born region and the ethnic admixture (the apparent ethnic phenotypes based on physical characteristics) are considered an element that helps the diagnosis of diseases. Such is the case of neuromyelitis optica and multiple sclerosis, which may be clinically similar but have well-established genetic susceptibility and ethnic differences. In Mexico, the susceptibility to neuromyelitis optica is given by HLA-DRB1^∗^16:02, and it usually occurs in individuals with a high Mexican Native American ancestry (Gorodezky et al., 1986; Romero-Hidalgo et al., 2020) (and usually, the physical characteristics indicate it). But in multiple sclerosis, the susceptibility is mainly given by HLA-DRB1^∗^15, and it occurs in individuals with higher Caucasian ancestry (Ordoñez et al., 2015). Another good example includes Multifocal Epithelial Hyperplasia, which depends on genetic susceptibility and is expressed in specific populations with high Mexican Native American components.

On the other hand, the technical aspect of this type of study is important. HLA sequencing study has helped to describe the ethnic admixture load in the patient and control populations. It deepens susceptibility and protection alleles and haplotypes due to high-fidelity performance. Finally, non-HLA variants in the MHC area contribute to the risk through linkage disequilibrium with HLA genes. Therefore, the CEHs help to explain better the susceptibility and protection in groups with known ethnic composition. However, the linkage of these variants with HLA requires a more in-depth analysis to understand how they add risk to CEH and the specific variants conserved in the Mexican population.

## Data Availability Statement

The datasets presented in this study can be found in the online repository: Allele frequency net database (AFND) with the accession Mexico Mexico City Mestizo population (*n* = 143) (SLE). African: Burkina Faso Mossi (*n* = 53), Cameroon Yaounde (*n* = 92), Ghana Ga-Adangbe (*n* = 141), Kenya (*n* = 144), Uganda Kampala pop 2 (*n* = 175), Kenya Nandi (*n* = 240), Zimbabwe Harare Shona (*n* = 230). European: England Blood Donors of Mixed Ethnicity (*n* = 519), France Lyon (*n* = 4813), Germany Essen (*n* = 174), Ireland Northern (*n* = 1000), Italy Lombardy (*n* = 674), Italy Sardinia pop 3 (*n* = 100), Netherlands UMCU (*n* = 64), Spain Catalunya, Navarra, Extremadura, Aragon, Cantabria (*n* = 4335). Mestizo Mexican populations from Northern Mexico: Mexico Baja California, Mexicali (*n* = 100), Mexico Chihuahua Chihuahua City (*n* = 119), Mexico Colima, Colima city (*n* = 61), Mexico Durango, Durango city (*n* = 153), Mexico Nuevo Leon, Monterrey city (*n* = 266), Mexico Sinaloa Rural (*n* = 183), Mexico Sonora Rural (*n* = 197). Mestizo Mexican populations from the Center of Mexico: Mexico Aguascalientes State (*n* = 95), Mexico Guanajuato Rural (*n* = 162), Mexico Guerrero state (*n* = 144), Mexico Jalisco, Guadalajara city (*n* = 1189), Mexico Mexico City Center (*n* = 152), Mexico Mexico City West (*n* = 33), Mexico Mexico City East (*n* = 79), Mexico Mexico City South (*n* = 52), Mexico Mexico City North (*n* = 751), Mexico Michoacan Rural (*n* = 348), Mexico Nayarit, Tepic (*n* = 97), Mexico Queretaro, Queretaro city (*n* = 45), Mexico San Luis Potosi Rural (*n* = 87), Mexico Mexico City Mestizo pop 2 (*n* = 234) (Controls). Mestizo Mexican populations from Southern Mexico: Mexico Chiapas Rural (*n* = 121), Mexico Oaxaca, Oaxaca city (*n* = 151), Mexico Quintana Roo Rural (*n* = 50), Mexico Tabasco Rural (*n* = 142), Native American Mexican populations: Mexico Nahuas (*n* = 72), Mexico Oaxaca Mixe (*n* = 55), Mexico Mixtec (*n* = 97), Mexico Chihuahua Tarahumara (*n* = 97), Mexico Oaxaca Mixe (*n* = 55), Mexico Oaxaca Mixtecs (*n* = 103), Mexico Oaxaca Zapotec (*n* = 90), Mexico Sonora Seri (*n* = 34), Mexico Chiapas Lacandon Mayans (*n* = 218). Non-Mexican Latin-American populations: Argentina Gran Chaco Eastern Toba (*n* = 125), Bolivia La Paz Aymaras (*n* = 88), Colombia Barranquilla (*n* = 188), Costa Rica African-Caribbean (*n* = 102), Costa Rica Amerindians (*n* = 125), Ecuador Andes Mixed Ancestry (*n* = 824), Nicaragua Managua (*n* = 339), Panama (*n* = 462). Asian: China Han (*n* = 314) http://www.allelefrequencies.net/ (Gonzalez-Galarza et al., 2020).

## Ethics Statement

The studies involving human participants were reviewed and approved by The Ethics Committee from the Instituto Nacional de Ciencias Médicas y Nutrición Salvador Zubirán. Reference No. 1738. The patients/participants provided their written informed consent to participate in this study.

## Author Contributions

SH-D, JG, GV-A, and JZ performed the conceptualization of the study. SH-D and JM-G performed the methodology. SH-D, JJ-O, LL, and GL investigated the study. JZ, JG, DR, VT-M, LL, and GL did the contribution with resources. SH-D, JM-G, and VA-A carried out the data curation. SH-D wrote the original draft and visualized the data. SH-D, JG, GV-A, and LL wrote, reviewed, and edited the draft of the manuscript. JZ, GV-A, and JG supervised the project. JG carried out the project administration. JG and JZ carried out the funding acquisition. All authors contributed to the article and approved the submitted version.

## Conflict of Interest

The authors declare that the research was conducted in the absence of any commercial or financial relationships that could be construed as a potential conflict of interest.

## Publisher’s Note

All claims expressed in this article are solely those of the authors and do not necessarily represent those of their affiliated organizations, or those of the publisher, the editors and the reviewers. Any product that may be evaluated in this article, or claim that may be made by its manufacturer, is not guaranteed or endorsed by the publisher.
