# Supplementary material for: Heterogeneity of Genetic Admixture Determines SLE Susceptibility in Mexican
Source: Front Genet. 2021 Aug 3;12:701373. doi: 10.3389/fgene.2021.701373 (PMC8369992; doi:10.3389/fgene.2021.701373)
Supplement: Supplementary file 1 [file Table_1.docx]

***Supplementary Table 1****.* HLA-A allele frequencies in SLE patients and healthy individuals.

| ***HLA-A alleles*** |  | **SLE** | |  | **Healthy individuals** | |  | ***pC*** | ***OR*** | ***95%IC*** | |
| --- | --- | --- | --- | --- | --- | --- | --- | --- | --- | --- | --- |
|  |  | N=143 (286 alleles) | |  | N=234 (468 alleles) | |  |  |  |  |  |
|  |  | ***n*** | ***AF*** |  | ***n*** | ***AF*** |  |  |  |  |  |
| A*02:01 |  | 58 | 0.2028 |  | 107 | 0.2286 |  | ns |  |  |  |
| A*24:02 |  | 41 | 0.1434 |  | 79 | 0.1688 |  | ns |  |  |  |
| **A*01:01** |  | **25** | **0.0874** |  | **17** | **0.0363** |  | **0.005** | **2.5** | **1.35** | **4.79** |
| A*31:01 |  | 20 | 0.0699 |  | 37 | 0.0791 |  | ns |  |  |  |
| A*02:06 |  | 17 | 0.0594 |  | 45 | 0.0962 |  | ns |  |  |  |
| A*68:01 |  | 14 | 0.0490 |  | 37 | 0.0791 |  | ns |  |  |  |
| A*03:01 |  | 15 | 0.0524 |  | 15 | 0.0321 |  | ns |  |  |  |
| **A*11:01** |  | **15** | **0.0524** |  | **10** | **0.0214** |  | **0.035** | **2.5** | **1.12** | **5.72** |
| A*29:02 |  | 11 | 0.0385 |  | 12 | 0.0256 |  | ns |  |  |  |
| A*68:03 |  | 12 | 0.0420 |  | 16 | 0.0342 |  | ns |  |  |  |
| A*68:02 |  | 8 | 0.0280 |  | 14 | 0.0299 |  | ns |  |  |  |
| A*74:01 |  | 1 | 0.0035 |  | 1 | 0.0021 |  | ns |  |  |  |
| A*32:01 |  | 4 | 0.0140 |  | 4 | 0.0085 |  | ns |  |  |  |
| A*23:01 |  | 3 | 0.0105 |  | 8 | 0.0171 |  | ns |  |  |  |
| A*26:01 |  | 3 | 0.0105 |  | 9 | 0.0192 |  | ns |  |  |  |
| A*30:01 |  | 3 | 0.0105 |  | 6 | 0.0128 |  | ns |  |  |  |
| A*68:05 |  | 2 | 0.0070 |  | 3 | 0.0064 |  | ns |  |  |  |
| A*33:03 |  | 2 | 0.0070 |  | 2 | 0.0043 |  | ns |  |  |  |
| A*33:01 |  | 2 | 0.0070 |  | 6 | 0.0128 |  | ns |  |  |  |
| A*34:02 |  | 2 | 0.0070 |  | 1 | 0.0021 |  | ns |  |  |  |
| A*02:02 |  | 1 | 0.0035 |  | 1 | 0.0021 |  | ns |  |  |  |
| A*02:05 |  | 1 | 0.0035 |  | 8 | 0.0171 |  | ns |  |  |  |
| A*66:01 |  | 1 | 0.0035 |  | 6 | 0.0128 |  | ns |  |  |  |
| A*30:02 |  | 8 | 0.0280 |  | 7 | 0.0150 |  | ns |  |  |  |
| Other alleles |  | 27 |  |  |  |  |  |  |  |  |  |
