# Supplementary material for: Heterogeneity of Genetic Admixture Determines SLE Susceptibility in Mexican
Source: Front Genet. 2021 Aug 3;12:701373. doi: 10.3389/fgene.2021.701373 (PMC8369992; doi:10.3389/fgene.2021.701373)
Supplement: Supplementary file 2 [file Table_2.docx]

***Supplementary Table 2****.* HLA-C allele frequencies in SLE patients and healthy individuals.

| **HLA-C alleles** |  | **SLE** | |  | **Healthy individuals** | |  | ***pC*** | ***OR*** | ***95%IC*** | |
| --- | --- | --- | --- | --- | --- | --- | --- | --- | --- | --- | --- |
|  |  | N=143 (286 alleles) | |  | N=234 (468 alleles) | |  |  |  |  |  |
|  |  | ***n*** | ***AF*** |  | ***n*** | ***AF*** |  |  |  |  |  |
| C*04:01 |  | 62 | 0.2168 |  | 87 | 0.1859 |  | ns |  |  |  |
| C*07:02 |  | 51 | 0.1783 |  | 97 | 0.2073 |  | ns |  |  |  |
| **C*07:01** |  | **31** | **0.1084** |  | **25** | **0.0534** |  | **0.008** | **2.2** | **1.24** | **3.73** |
| C*03:04 |  | 20 | 0.0699 |  | 31 | 0.0662 |  | ns |  |  |  |
| C*01:02 |  | 16 | 0.0559 |  | 42 | 0.0897 |  | ns |  |  |  |
| C*16:01 |  | 12 | 0.0420 |  | 12 | 0.0256 |  | ns |  |  |  |
| C*08:02 |  | 10 | 0.0350 |  | 19 | 0.0406 |  | ns |  |  |  |
| C*08:01 |  | 11 | 0.0385 |  | 22 | 0.0470 |  | ns |  |  |  |
| C*06:02 |  | 10 | 0.0350 |  | 28 | 0.0598 |  | ns |  |  |  |
| C*03:03 |  | 9 | 0.0315 |  | 14 | 0.0299 |  | ns |  |  |  |
| C*05:01 |  | 7 | 0.0245 |  | 10 | 0.0214 |  | ns |  |  |  |
| C*12:03 |  | 6 | 0.0210 |  | 12 | 0.0256 |  | ns |  |  |  |
| C*14:02 |  | 8 | 0.0280 |  | 4 | 0.0085 |  | ns |  |  |  |
| C*02:10 |  | 5 | 0.0175 |  | 2 | 0.0043 |  | ns |  |  |  |
| C*17:01 |  | 3 | 0.0105 |  | 1 | 0.0021 |  | ns |  |  |  |
| C*02:02 |  | 2 | 0.0070 |  | 6 | 0.0128 |  | ns |  |  |  |
| C*12:02 |  | 2 | 0.0070 |  | 2 | 0.0043 |  | ns |  |  |  |
| C*15:09 |  | 2 | 0.0070 |  | 11 | 0.0235 |  | ns |  |  |  |
| C*07:04 |  | 2 | 0.0070 |  | 1 | 0.0021 |  | ns |  |  |  |
| C*15:02 |  | 2 | 0.0070 |  | 9 | 0.0192 |  | ns |  |  |  |
| C*03:02 |  | 2 | 0.0070 |  | 2 | 0.0043 |  | ns |  |  |  |
| C*04:07 |  | 1 | 0.0035 |  | 2 | 0.0043 |  | ns |  |  |  |
| C*08:03 |  | 1 | 0.0035 |  | 4 | 0.0085 |  | ns |  |  |  |
| C*15:05 |  | 1 | 0.0035 |  | 1 | 0.0021 |  | ns |  |  |  |
| C*16:02 |  | 1 | 0.0035 |  | 1 | 0.0021 |  | ns |  |  |  |
| Other alleles |  | 9 |  |  |  |  |  |  |  |  |  |
