# Supplementary material for: Heterogeneity of Genetic Admixture Determines SLE Susceptibility in Mexican
Source: Front Genet. 2021 Aug 3;12:701373. doi: 10.3389/fgene.2021.701373 (PMC8369992; doi:10.3389/fgene.2021.701373)
Supplement: Supplementary file 3 [file Table_3.docx]

***Supplementary Table 3*.** HLA-DQB1 allele frequencies in SLE patients and healthy individuals.

| **HLA-DQB1 alleles** |  | **SLE** | |  | **Healthy individuals** | |  | ***pC*** | ***OR*** | ***95%IC*** | |
| --- | --- | --- | --- | --- | --- | --- | --- | --- | --- | --- | --- |
|  |  | N=143 (286 alleles) | |  | N=234 (468 alleles) | |  |  |  |  |  |
|  |  | ***n*** | ***AF*** |  | ***n*** | ***AF*** |  |  |  |  |  |
| DQB1*03:02 |  | 60 | 0.2098 |  | 115 | 0.2457 |  | ns |  |  |  |
| DQB1*04:02 |  | 59 | 0.2063 |  | 96 | 0.2051 |  | ns |  |  |  |
| **DQB1*03:01** |  | **42** | **0.1469** |  | **116** | **0.2479** |  | **0.001** | **0.5** | **0.35** | **0.77** |
| **DQB1*02:01** |  | **29** | **0.1014** |  | **15** | **0.0321** |  | **0.0002** | **3.4** | **1.79** | **6.47** |
| DQB1*02:02 |  | 25 | 0.0874 |  | 28 | 0.0598 |  | ns |  |  |  |
| **DQB1*06:02** |  | **22** | **0.0769** |  | **17** | **0.0363** |  | **0.02** | **2.2** | **1.15** | **4.24** |
| DQB1*05:01 |  | 21 | 0.0734 |  | 32 | 0.0684 |  | ns |  |  |  |
| DQB1*06:03 |  | 8 | 0.0280 |  | 7 | 0.0150 |  | ns |  |  |  |
| DQB1*03:19 |  | 4 | 0.0140 |  | 5 | 0.0107 |  | ns |  |  |  |
| DQB1*03:03 |  | 3 | 0.0105 |  | 10 | 0.0214 |  | ns |  |  |  |
| DQB1*03:04 |  | 2 | 0.0070 |  | 0 | 0.0000 |  | ns |  |  |  |
| DQB1*05:02 |  | 2 | 0.0070 |  | 3 | 0.0064 |  | ns |  |  |  |
| DQB1*05:03 |  | 2 | 0.0070 |  | 8 | 0.0171 |  | ns |  |  |  |
| DQB1*06:04 |  | 1 | 0.0035 |  | 10 | 0.0214 |  | ns |  |  |  |
| DQB1*06:09 |  | 1 | 0.0035 |  | 0 | 0.0000 |  | ns |  |  |  |
| Other alleles |  | 5 |  |  |  |  |  |  |  |  |  |
