# Supplementary material for: Heterogeneity of Genetic Admixture Determines SLE Susceptibility in Mexican
Source: Front Genet. 2021 Aug 3;12:701373. doi: 10.3389/fgene.2021.701373 (PMC8369992; doi:10.3389/fgene.2021.701373)
Supplement: Supplementary file 4 [file Table_4.docx]

**Table S4.** Frequencies of HLA-C/-B haplotypes in SLE patients and healthy individuals.

| **HLA-C/-B Haplotypes** |  | **SLE N=286** | | |  | **Healthy Individuals N=468** | | |  | **pC** | **OR** | **95%IC** | |
| --- | --- | --- | --- | --- | --- | --- | --- | --- | --- | --- | --- | --- | --- |
|  |  | **n** | **HF** | **Δ'** |  | **n** | **HF** | **Δ'** |  |  |  |  |  |
| **African** | | | | | | | | | | | | | |
| C*02:10~B*15:03 |  | 5 | 0.0175 | 1.000 |  | 1 | 0.0021 | 0.4978 |  | ns |  |  |  |
| C*14:02~B*15:16 |  | 2 | 0.0070 | 1.000 |  | 1 | 0.0021 | 1.0000 |  | ns |  |  |  |
| C*04:01~B*53:01 |  | 1 | 0.0035 | 1.000 |  | 6 | 0.0128 | 1.0000 |  | ns |  |  |  |
| C*07:01~B*57:01 |  | 1 | 0.0035 | 0.438 |  | 3 | 0.0064 | 0.3960 |  | ns |  |  |  |
| C*16:01~B*51:01 |  | 1 | 0.0035 | 0.028 |  | 1 | 0.0021 | 0.0245 |  | ns |  |  |  |
| **Amerindian** | | | | | | | | | | | | | |
| C*07:02~B*39:05 |  | 26 | 0.0909 | 1.000 |  | 34 | 0.0726 | 0.8975 |  | ns |  |  |  |
| C*04:01~B*35:17 |  | 15 | 0.0524 | 1.000 |  | 17 | 0.0363 | 1.0000 |  | ns |  |  |  |
| C*04:01~B*35:12 |  | 13 | 0.0455 | 1.000 |  | 16 | 0.0342 | 0.8632 |  | ns |  |  |  |
| C*03:04~B*40:02 |  | 9 | 0.0315 | 0.892 |  | 11 | 0.0235 | 0.4196 |  | ns |  |  |  |
| C*07:02~B*39:06 |  | 8 | 0.0280 | 1.000 |  | 29 | 0.0620 | 0.8025 |  | ns |  |  |  |
| C*08:01~B*48:01 |  | 8 | 0.0280 | 0.884 |  | 15 | 0.0321 | 0.7376 |  | ns |  |  |  |
| C*01:02~B*15:01 |  | 3 | 0.0105 | 0.576 |  | 7 | 0.0150 | 0.6701 |  | ns |  |  |  |
| C*03:04~B*35:01 |  | 2 | 0.0070 | 0.037 |  | 1 | 0.0021 | -0.4456 |  | ns |  |  |  |
| C*07:01~B*15:17 |  | 2 | 0.0070 | 1.000 |  | 3 | 0.0064 | 1.0000 |  | ns |  |  |  |
| C*03:04~B*40:05 |  | 1 | 0.0035 | 1.000 |  | 3 | 0.0064 | 0.5714 |  | ns |  |  |  |
| C*04:01~B*35:03 |  | 1 | 0.0035 | 1.000 |  | 4 | 0.0085 | 0.7538 |  | ns |  |  |  |
| C*08:01~B*51:02 |  | 1 | 0.0035 | 1.000 |  | 2 | 0.0043 | 1.0000 |  | ns |  |  |  |
| C*08:03~B*48:01 |  | 1 | 0.0035 | 1.000 |  | 3 | 0.0064 | 0.7387 |  | ns |  |  |  |
| C*07:02~B*39:01 |  | 3 | 0.0105 | 1.000 |  | 4 | 0.0085 | 0.7471 |  | ns |  |  |  |
| C*01:02~B*15:30 |  | 2 | 0.0070 | 1.000 |  | 8 | 0.0171 | 1.0000 |  | ns |  |  |  |
| **Asian** | | | | | | | | | | | | | |
| C*03:02~B*58:01 |  | 2 | 0.0070 | 1.000 |  | 1 | 0.0021 | 0.4967 |  | ns |  |  |  |
| C*04:01~B*35:16 |  | 2 | 0.0070 | 1.000 |  | 3 | 0.0064 | 1.0000 |  | ns |  |  |  |
| C*03:03~B*35:01 |  | 1 | 0.0035 | 0.047 |  | 1 | 0.0021 | 0.0141 |  | ns |  |  |  |
| C*14:02~B*51:01 |  | 0 | 0.0000 | 0.735 |  | 3 | 0.0064 | 0.7339 |  | ns |  |  |  |
| **Caucasian** | | | | | | | | | | | | | |
| **C*07:01~B*08:01** |  | **19** | **0.0664** | 0.944 |  | **3** | **0.0064** | **1.0000** |  | **0.000006** | **11.0** | **3.23** | **37.62** |
| C*07:02~B*07:02 |  | 11 | 0.0385 | 0.812 |  | 15 | 0.0321 | 0.7893 |  | ns |  |  |  |
| C*16:01~B*44:03 |  | 10 | 0.0350 | 0.825 |  | 8 | 0.0171 | 0.6571 |  | ns |  |  |  |
| C*05:01~B*18:01 |  | 4 | 0.0140 | 0.556 |  | 5 | 0.0107 | 0.6167 |  | ns |  |  |  |
| C*06:02~B*37:01 |  | 4 | 0.0140 | 1.000 |  | 3 | 0.0064 | 0.7339 |  | ns |  |  |  |
| C*04:01~B*44:03 |  | 3 | 0.0105 | 0.014 |  | 3 | 0.0064 | 0.0533 |  | ns |  |  |  |
| C*12:03~B*38:01 |  | 3 | 0.0105 | 0.745 |  | 6 | 0.0128 | 1.0000 |  | ns |  |  |  |
| C*05:01~B*44:02 |  | 2 | 0.0070 | 1.000 |  | 4 | 0.0085 | 0.7956 |  | ns |  |  |  |
| C*06:02~B*50:01 |  | 2 | 0.0070 | 1.000 |  | 4 | 0.0085 | 1.0000 |  | ns |  |  |  |
| C*12:03~B*18:01 |  | 2 | 0.0070 | 0.378 |  | 2 | 0.0043 | 0.2301 |  | ns |  |  |  |
| C*03:04~B*15:01 |  | 1 | 0.0035 | 0.139 |  | 2 | 0.0043 | 0.1427 |  | ns |  |  |  |
| C*06:02~B*57:01 |  | 1 | 0.0035 | 0.482 |  | 2 | 0.0043 | 0.2398 |  | ns |  |  |  |
| **Caucasian shared with other population** | | | | | | | | | | | | | |
| C*04:01~B*35:01 |  | 15 | 0.0524 | 0.730 |  | 15 | 0.0321 | 0.4530 |  | ns |  |  |  |
| C*08:02~B*14:02 |  | 7 | 0.0245 | 1.000 |  | 11 | 0.0235 | 0.7219 |  | ns |  |  |  |
| C*15:02~B*51:01 |  | 7 | 0.0245 | 0.470 |  | 9 | 0.0192 | 1.0000 |  | ns |  |  |  |
| C*07:01~B*49:01 |  | 4 | 0.0140 | 1.000 |  | 6 | 0.0128 | 0.6477 |  | ns |  |  |  |
| C*08:02~B*14:01 |  | 3 | 0.0105 | 1.000 |  | 4 | 0.0085 | 1.0000 |  | ns |  |  |  |
| C*06:02~B*13:02 |  | 2 | 0.0070 | 1.000 |  | 5 | 0.0107 | 0.8226 |  | ns |  |  |  |
| C*12:02~B*52:01 |  | 2 | 0.0070 | 1.000 |  | 2 | 0.0043 | 1.0000 |  | ns |  |  |  |
| C*02:02~B*40:02 |  | 1 | 0.0035 | 0.482 |  | 1 | 0.0021 | 0.1212 |  | ns |  |  |  |
| C*07:01~B*41:01 |  | 1 | 0.0035 | 0.438 |  | 4 | 0.0085 | 0.7886 |  | ns |  |  |  |
| **Unknown** | | | | | | | | | | | | | |
| C*03:03~B*52:01 |  | 7 | 0.0245 | 0.742 |  | 6 | 0.0128 | 0.5876 |  | ns |  |  |  |
| C*01:02~B*15:15 |  | 6 | 0.0210 | 1.000 |  | 13 | 0.0278 | 0.8534 |  | ns |  |  |  |
| C*01:02~B*35:43 |  | 3 | 0.0105 | 1.000 |  | 9 | 0.0192 | 1.0000 |  | ns |  |  |  |
| C*04:01~B*35:14 |  | 3 | 0.0105 | 1.000 |  | 6 | 0.0128 | 1.0000 |  | ns |  |  |  |
| C*15:09~B*51:01 |  | 2 | 0.0070 | 1.000 |  | 9 | 0.0192 | 0.8065 |  | ns |  |  |  |
| C*03:03~B*15:01 |  | 1 | 0.0035 | 0.174 |  | 1 | 0.0021 | 0.0720 |  | ns |  |  |  |
| C*03:04~B*40:20 |  | 1 | 0.0035 | 1.000 |  | 1 | 0.0021 | 1.0000 |  | ns |  |  |  |
| C*04:01~B*35:08 |  | 1 | 0.0035 | 1.000 |  | 3 | 0.0064 | 1.0000 |  | ns |  |  |  |
| C*04:07~B*15:31 |  | 1 | 0.0035 | 1.000 |  | 2 | 0.0043 | 1.0000 |  | ns |  |  |  |
| C*08:01~B*51:01 |  | 1 | 0.0035 | 0.036 |  | 1 | 0.0021 | -0.2468 |  | ns |  |  |  |
