# Supplementary material for: Heterogeneity of Genetic Admixture Determines SLE Susceptibility in Mexican
Source: Front Genet. 2021 Aug 3;12:701373. doi: 10.3389/fgene.2021.701373 (PMC8369992; doi:10.3389/fgene.2021.701373)
Supplement: Supplementary file 5 [file Data_Sheet_1.docx]

1. ***Supplementary information***

***Supplementary Information 1 (Population average pairwise differences, Matrix color showed in the article as Figure 2)***

GENETIC STRUCTURE ANALYSIS
Comparisons of pairs of population samples
List of labels for population samples used below:
-------------------------------------------------
Label Population name
----- ---------------
 1: SLE
 2: Healthy Individuals
 3: Spaniards
 4: Tarahumaras
 5: Seri
 6: Mixtecs
 7: Zapotecans
 8: Mixe
 9: Lacandon
 10: Zimbabwe Harare
 11: China Han

---------------------------------------
**Population average pairwise differences**----------------------------------------

Above diagonal: Average number of pairwise differences between populations (PiXY)
Diagonal elements: Average number of pairwise differences within population (PiX)
Below diagonal: Corrected average pairwise difference (PiXY-(PiX+PiY)/2)

Computing conventional F-Statistics from haplotype frequencies
</data><pairwiseDifferenceMatrix time="09-12-20 at 13-24-20" graphicExist="yes">

 1 2 3 4 5 6 7 8 9 10 11
 1 0.96506 0.96547 0.97151 0.97483 0.95155 0.95994 0.95809 0.96986 0.95288 0.98404 0.98981
 2 **0.00204** 0.96180 0.97612 0.96714 0.94866 0.95443 0.95555 0.96289 0.94804 0.98881 0.98993
 3 0.01275 0.01899 0.95246 0.98158 0.96188 0.99166 0.98369 0.99218 0.98044 0.97549 0.98393
 4 0.05474 0.04867 0.06779 0.87513 0.87941 0.97311 0.95257 0.96159 0.91410 0.99691 0.98284
 5 0.10945 0.10819 0.12609 0.08228 0.71913 0.96269 0.87998 0.93204 0.85290 0.99087 0.97913
 6 0.02642 0.02254 0.06444 0.08456 0.15213 0.90198 0.93965 0.95907 0.93548 0.99766 0.99484
 7 0.02417 0.02326 0.05607 0.06362 0.06902 0.03727 0.90278 0.90139 0.91009 0.99412 0.99016
 8 0.09592 0.09058 0.12455 0.13262 0.18106 0.11667 0.05859 0.78282 0.91625 0.99844 0.99567
 9 0.05046 0.04724 0.08432 0.05664 0.07344 0.06460 0.03881 0.10495 0.83979 0.99346 0.99048
 10 0.03027 0.03667 0.02802 0.08810 0.16006 0.07543 0.07149 0.13579 0.10233 0.94248 0.99214
 11 0.04014 0.04189 0.04056 0.07813 0.15242 0.07670 0.07162 0.13712 0.10345 0.05376 0.93429
</pairwiseDifferenceMatrix><data>

-----------
**PXY P-value**-----------

 1 2 3 4 5 6 7 8 9 10

 2 **0.00909**
 3 0.00000 0.00000
 4 0.00000 0.00000 0.00000
 5 0.56364 0.92727 0.00000 0.00000
 6 0.00000 0.14545 0.00000 0.00000 0.00000
 7 0.02727 0.50000 0.00000 0.00000 0.29091 0.00000
 8 0.00000 0.00000 0.00000 0.00000 0.00000 0.00000 0.00000
 9 0.00000 0.00000 0.00000 0.00000 0.10909 0.00000 0.00000 0.00000
 10 0.00000 0.00000 0.00000 0.00000 0.00000 0.00000 0.00000 0.00000 0.00000
 11 0.00000 0.00000 0.00000 0.00000 0.00000 0.00000 0.00000 0.00000 0.00000 0.00000


---------------------
**Corrected PXY P-value**---------------------

 1 2 3 4 5 6 7 8 9 10

 2 **0.01818**
 3 0.00000 0.00000
 4 0.00000 0.00000 0.00000
 5 0.00000 0.00000 0.00000 0.00000
 6 0.00000 0.00000 0.00000 0.00000 0.00000
 7 0.00000 0.00000 0.00000 0.00000 0.00000 0.00000
 8 0.00000 0.00000 0.00000 0.00000 0.00000 0.00000 0.00000
 9 0.00000 0.00000 0.00000 0.00000 0.00000 0.00000 0.00000 0.00000
 10 0.00000 0.00000 0.00000 0.00000 0.00000 0.00000 0.00000 0.00000 0.00000
 11 0.00000 0.00000 0.00000 0.00000 0.00000 0.00000 0.00000 0.00000 0.00000 0.00000
