# Supplementary material for: Heterogeneity of Genetic Admixture Determines SLE Susceptibility in Mexican
Source: Front Genet. 2021 Aug 3;12:701373. doi: 10.3389/fgene.2021.701373 (PMC8369992; doi:10.3389/fgene.2021.701373)
Supplement: Supplementary file 6 [file Image_1.pdf]

Supplementary Material

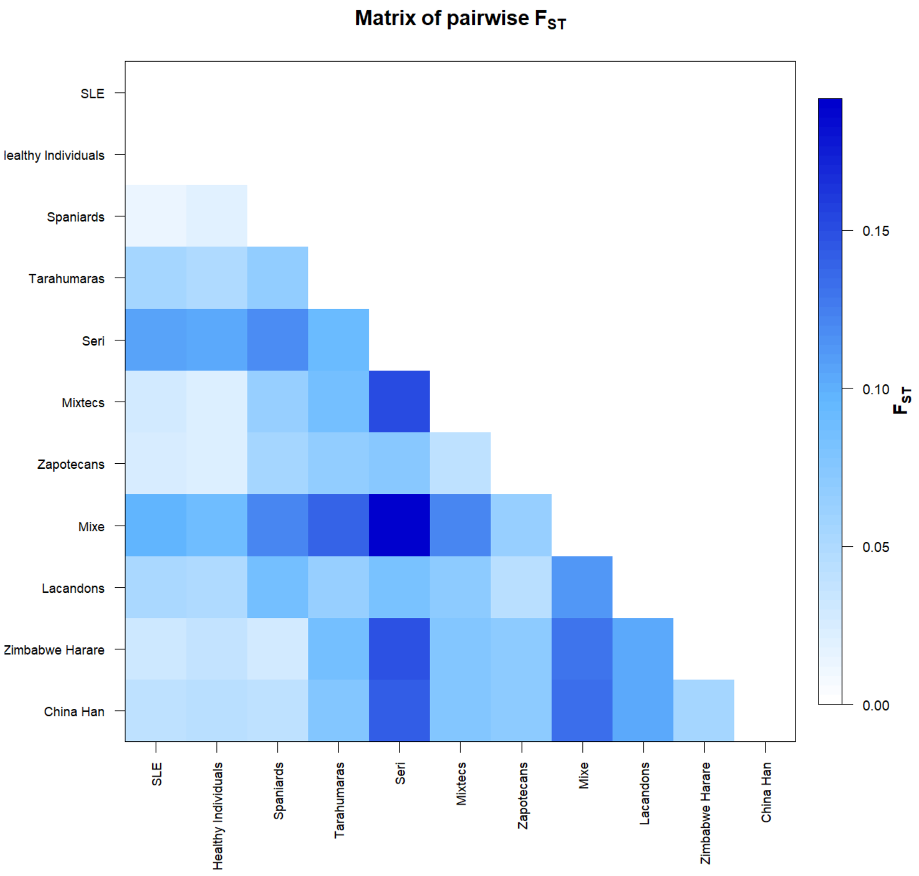

Supplementary Figure 1. Populations pairwise FSTs

GENETIC STRUCTURE ANALYSIS
Comparisons of pairs of population samples
List of labels for population samples used below:

Label	Population name
-----	---------------
1:	SLE
2:	Healthy Individuals
3:	Spaniards
4:	Tarahumaras
5:	Seri
6:	Mixtecs
7:	Zapotecans
8:	Mixe
9:	Lacandon
10:	Zimbabwe Harare
11:	China Han

Population pairwise FSTs

Computing conventional F-Statistics from haplotype frequencies

	1	2	3	4	5	6	7	8	9	10	11
1	0.00000										
2	**0.00211**	0.00000									
3	0.01316	0.01946	0.00000								
4	0.05447	0.04849	0.06688	0.00000							
5	0.10524	0.10291	0.11871	0.09215	0.00000						
6	0.02730	0.02324	0.06419	0.08636	0.15024	0.00000					
7	0.02485	0.02383	0.05611	0.06649	0.07479	0.03966	0.00000				
8	0.09584	0.08963	0.11970	0.13987	0.19151	0.12060	0.06511	0.00000			
9	0.05378	0.04984	0.08569	0.06293	0.08106	0.07004	0.04356	0.11271	0.00000		
10	0.03086	0.03709	0.02871	0.08612	0.14787	0.07493	0.07109	0.13040	0.10288	0.00000	
11	0.04076	0.04237	0.04123	0.07756	0.14229	0.07648	0.07161	0.13180	0.10395	0.05420	0.00000

FST P values

Number of permutations: 110

	1	2	3	4	5	6	7	8	9	10	11
1	*										
2	**0.01802+-0.0121**	*									
3	0.00000+-0.0000	0.00000+-0.0000	*								
4	0.00000+-0.0000	0.00000+-0.0000	0.00000+-0.0000	*							
5	0.00000+-0.0000	0.00000+-0.0000	0.00000+-0.0000	0.00000+-0.0000	*						
6	0.00000+-0.0000	0.00000+-0.0000	0.00000+-0.0000	0.00000+-0.0000	0.00000+-0.0000	*					
7	0.00000+-0.0000	0.00000+-0.0000	0.00000+-0.0000	0.00000+-0.0000	0.00000+-0.0000	0.00000+-0.0000	*				
8	0.00000+-0.0000	0.00000+-0.0000	0.00000+-0.0000	0.00000+-0.0000	0.00000+-0.0000	0.00000+-0.0000	0.00000+-0.0000	*			
9	0.00000+-0.0000	0.00000+-0.0000	0.00000+-0.0000	0.00000+-0.0000	0.00000+-0.0000	0.00000+-0.0000	0.00000+-0.0000	0.00000+-0.0000	*		
10	0.00000+-0.0000	0.00000+-0.0000	0.00000+-0.0000	0.00000+-0.0000	0.00000+-0.0000	0.00000+-0.0000	0.00000+-0.0000	0.00000+-0.0000	0.00000+-0.0000	*	
11	0.00000+-0.0000	0.00000+-0.0000	0.00000+-0.0000	0.00000+-0.0000	0.00000+-0.0000	0.00000+-0.0000	0.00000+-0.0000	0.00000+-0.0000	0.00000+-0.0000	0.00000+-0.0000	*

Matrix of significant Fst P values

Significance Level=0.0500

Number of permutations: 110

	1	2	3	4	5	6	7	8	9	10	11
1		+	+	+	+	+	+	+	+	+	+
2	+		+	+	+	+	+	+	+	+	+
3	+	+		+	+	+	+	+	+	+	+
4	+	+	+		+	+	+	+	+	+	+
5	+	+	+	+		+	+	+	+	+	+
6	+	+	+	+	+		+	+	+	+	+
7	+	+	+	+	+	+		+	+	+	+
8	+	+	+	+	+	+	+		+	+	+
9	+	+	+	+	+	+	+	+		+	+
10	+	+	+	+	+	+	+	+	+		+
11	+	+	+	+	+	+	+	+	+	+	
