# Supplementary material for: Heterogeneity of Genetic Admixture Determines SLE Susceptibility in Mexican
Source: Front Genet. 2021 Aug 3;12:701373. doi: 10.3389/fgene.2021.701373 (PMC8369992; doi:10.3389/fgene.2021.701373)
Supplement: Supplementary file 7 [file Image_2.pdf]

Supplementary Material

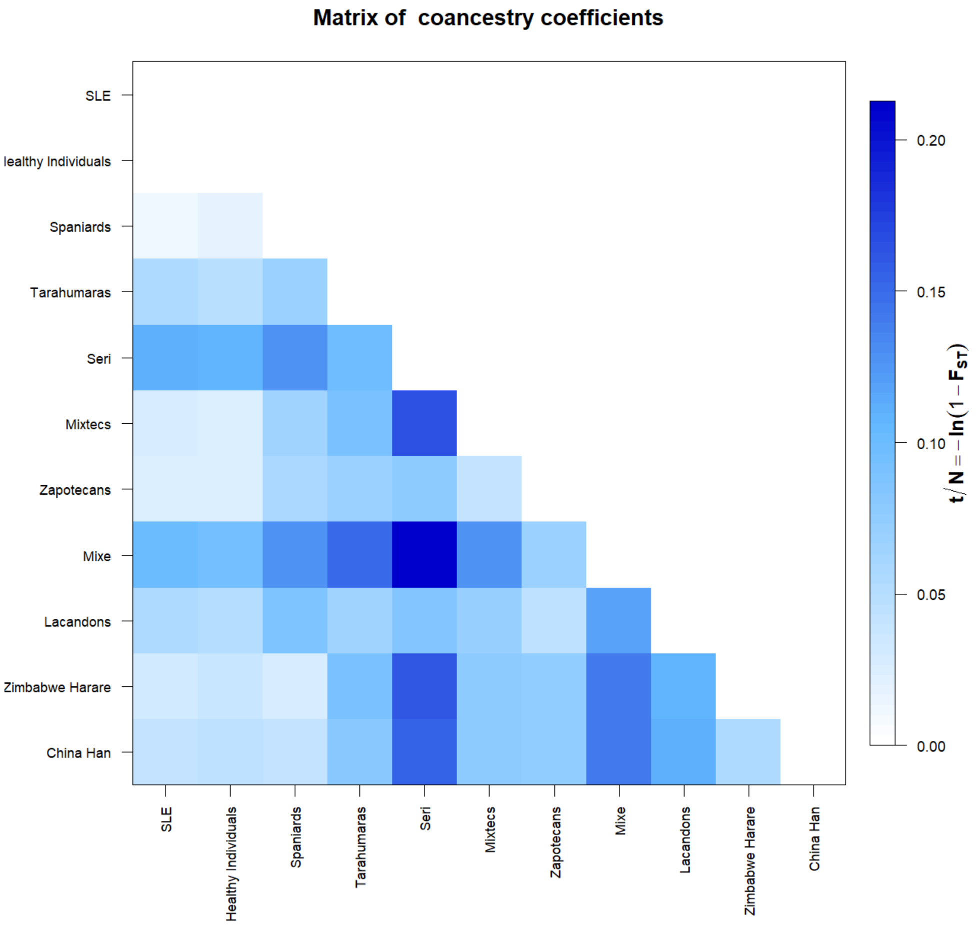

Supplementary Figure 2. Matrix of Coancestry coefficients

GENETIC STRUCTURE ANALYSIS
Comparisons of pairs of population samples
List of labels for population samples used below:

Label	Population name
-----	---------------
1:	SLE
2:	Healthy Individuals
3:	Spaniards
4:	Tarahumaras
5:	Seri
6:	Mixtecs
7:	Zapotecans
8:	Mixe
9:	Lacandon
10:	Zimbabwe Harare
11:	China Han

Matrix of coancestry coefficients as t/M=-ln(1-FST)
(M=N for haploid data, M=2N for diploid data)

Reference: </data><Reference>Reynolds, J., Weir, B.S., and Cockerham, C.C. 1983.</Reference><data>
</data><coancestryCoefficients time="09-12-20 at 13-24-20" graphicExist="yes">

	1	2	3	4	5	6	7	8	9	10	11
1	-0.00000										
2	**0.00212**	-0.00000									
3	0.01324	0.01966	-0.00000								
4	0.05601	0.04971	0.06922	-0.00000							
5	0.11120	0.10860	0.12637	0.09668	-0.00000						
6	0.02768	0.02352	0.06634	0.09032	0.16280	-0.00000					
7	0.02517	0.02412	0.05774	0.06880	0.07773	0.04047	-0.00000				
8	0.10075	0.09391	0.12750	0.15067	0.21259	0.12852	0.06732	-0.00000			
9	0.05528	0.05112	0.08958	0.06500	0.08454	0.07261	0.04453	0.11958	-0.00000		
10	0.03134	0.03780	0.02913	0.09005	0.16001	0.07789	0.07374	0.13973	0.10856	-0.00000	
11	0.04162	0.04330	0.04211	0.08073	0.15349	0.07956	0.07430	0.14134	0.10976	0.05573	-0.00000

</coancestryCoefficients><data>
