# Supplementary material for: Heterogeneity of Genetic Admixture Determines SLE Susceptibility in Mexican
Source: Front Genet. 2021 Aug 3;12:701373. doi: 10.3389/fgene.2021.701373 (PMC8369992; doi:10.3389/fgene.2021.701373)
Supplement: Supplementary file 8 [file Image_3.pdf]

Supplementary Material

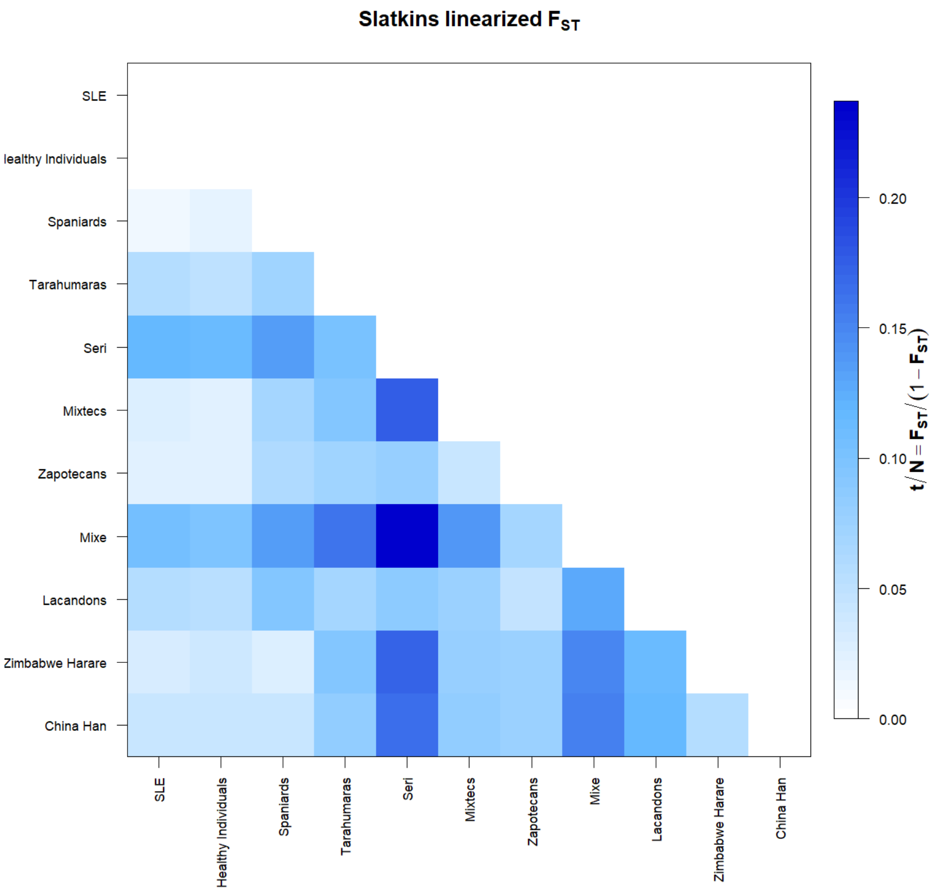

Supplementary Figure 3. Slatkin's linearized FST

GENETIC STRUCTURE ANALYSIS

Comparisons of pairs of population samples

List of labels for population samples used below:

Label	Population name
-----	---------------
1:	SLE
2:	Healthy Individuals
3:	Spaniards
4:	Tarahumaras
5:	Seri
6:	Mixtecs
7:	Zapotecans
8:	Mixe
9:	Lacandon
10:	Zimbabwe Harare
11:	China Han

Matrix of Slatkin linearized FSTs as t/M=FST/(1-FST)
(M=N for haploid data, M=2N for diploid data)

Reference: </data><Reference>Slatkin, M., 1995. </Reference><data>
</data><slatkinFst time="09-12-20 at 13-24-20" graphicExist="yes">

	1	2	3	4	5	6	7	8	9	10	11
1	0.00000										
2	**0.00212**	0.00000									
3	0.01333	0.01985	0.00000								
4	0.05761	0.05097	0.07167	0.00000							
5	0.11762	0.11472	0.13470	0.10150	0.00000						
6	0.02807	0.02380	0.06860	0.09453	0.17680	0.00000					
7	0.02549	0.02441	0.05944	0.07123	0.08083	0.04130	0.00000				
8	0.10600	0.09846	0.13598	0.16262	0.23688	0.13714	0.06964	0.00000			
9	0.05684	0.05245	0.09372	0.06716	0.08822	0.07532	0.04554	0.12702	0.00000		
10	0.03184	0.03852	0.02956	0.09423	0.17353	0.08100	0.07653	0.14996	0.11467	0 .00000	
11	0.04250	0.04425	0.04301	0.08408	0.16590	0.08281	0.07713	0.15181	0.11601	0 .05731	0.00000

</slatkinFst><data>
