# Supplementary material for: Heterogeneity of Genetic Admixture Determines SLE Susceptibility in Mexican
Source: Front Genet. 2021 Aug 3;12:701373. doi: 10.3389/fgene.2021.701373 (PMC8369992; doi:10.3389/fgene.2021.701373)
Supplement: Supplementary file 9 [file Image_4.pdf]

Supplementary Material

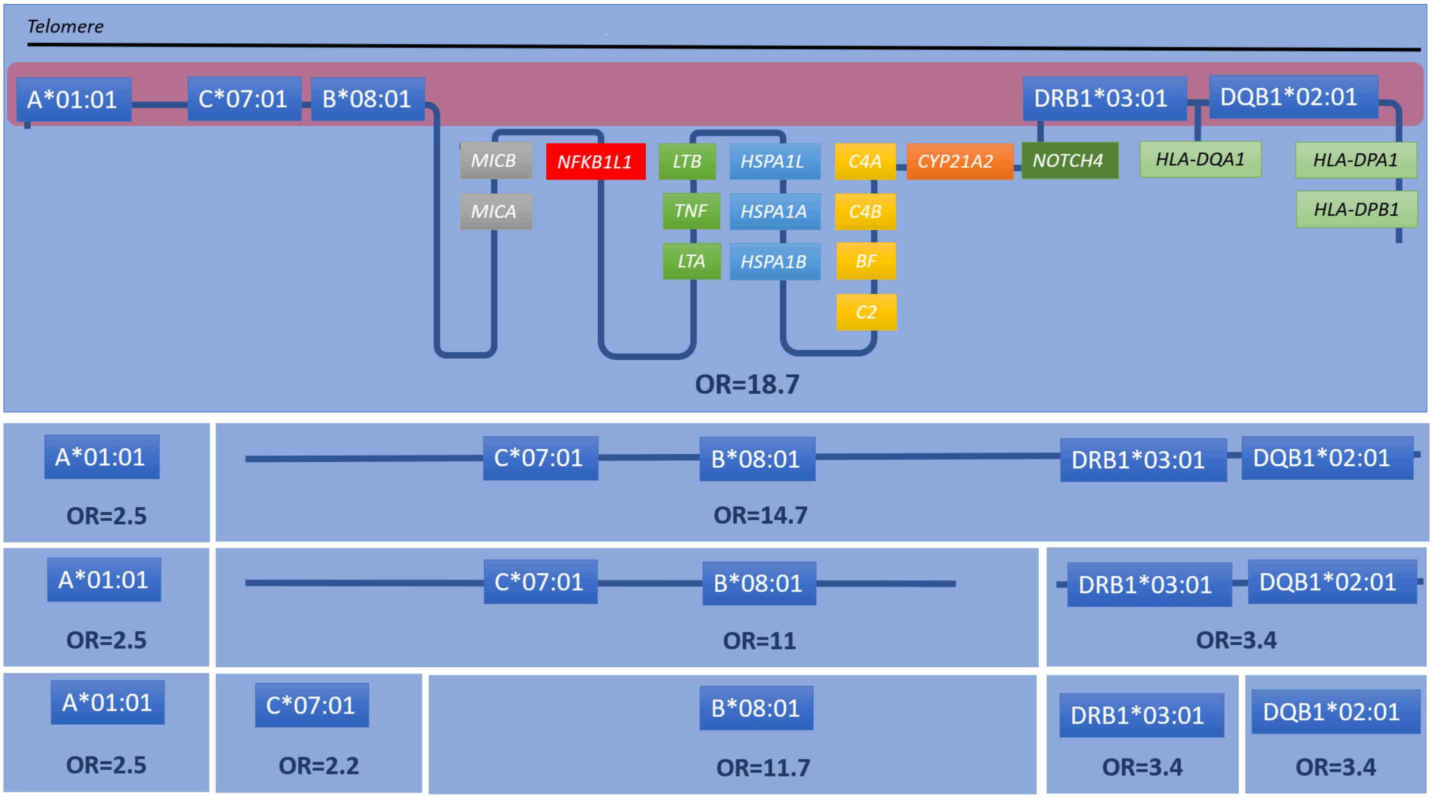

Supplementary Figure 4. The conserved extended susceptibility haplotype most common in SLE Mexican mestizo patients. The linkage disequilibrium effect on the haplotype: A*01:01~C*07:01-B*08:01 ~DRB1*03:01~DQB1*02:01, is disclosed in the figure. This Conserved Extended Haplotype has a higher relative risk (OR), which partly proves that the linkage disequilibrium influences the heritage block of the HLA variants (Pink Band). The linkage disequilibrium with MHC class III variants and non-HLA gives a complex genetic load to this genetic segment.

This phenomenon is observable in the relative risks in partial blocks, including B*08:01 and DRB1*03:01. Many MHC Class III gene polymorphisms have been associated with SLE in independent studies in Mexicans. The linkage of the MHC Class III specific variants and the rest of HLA alleles have been corroborated in Spaniards. Still, they need to be verified in Mexicans and conclude about MHC complete area's influence on the different SLE phenotypes in both populations.

Abbreviations: steroid 21-hydroxylase (CYP21A2); factor B (BF); three 70 kDa heat-shock proteins (HSPA1A, HSPA1B, HSPA1L); natural cytotoxicity triggering receptor 3 (NCR3, also known as 1C7); nuclear factor kappa light chain gene enhancer in B cells inhibitor-like 1 (NFKBIL1); proto-oncogene NOTCH4 (Notch homologue 4). The figure is not at scale.
